# Soluble Electrochromic Polymers Incorporating Benzoselenadiazole and Electron Donor Units (Carbazole or Fluorene): Synthesis and Electronic-Optical Properties

**DOI:** 10.3390/polym10040450

**Published:** 2018-04-17

**Authors:** Jianzhong Xu, Qi Ji, Lingqian Kong, Hongmei Du, Xiuping Ju, Jinsheng Zhao

**Affiliations:** 1 College of Chemistry and Environmental Science, Hebei University, Baoding 071002, China; xujianzhong@126.com (J.X.); wangyan@lcu.edu.cn (Q.J.); 2 Department of Chemistry and Chemical Engineering, Liaocheng University, Liaocheng 252059, China; duhongmei@lcu.edu.cn; 3 Dongchang Colledge, Liaocheng University, Liaocheng 252059, China; lingqiankong@126.com (L.K.); jxp1127@163.com (X.J.)

**Keywords:** benzoselenadiazole, carbazole, fluorene, conjugated polymer, electrochromic, solution processable

## Abstract

A series of π-conjugated polymers containing alternating benzoselenadiazole (BSe)-bi(thiophene derivative)-carbazole or benzoththiadiazole (BSe)-bi(thiophene derivative)-fluorene units were designed and synthesized. Thiophene derivatives, namely 3-hexylthiophene, 3,4-bihexyloxythiophene, and 3,4-bioctyloxythiophene, were used as the π-bridges of the polymers. The polymers were characterized in detail in terms of their thermal stabilities, cyclic voltammograms, UV-Vis absorption, spectroelectrochemistry, dynamic switching property and so forth. The alkoxy thiophene π-bridged polymers have lower onset oxidation potentials and bandgaps than that of their corresponding alkyl thiophene π-bridged polymers. The selection of the donor units between the carbazole and the fluorene units has nearly no effect on the bandgaps and colors as well as the onset oxidation potentials of the polymers. The increase in the length of the side alkyl chains on the thiophene ring caused a slight increase in the polymer bandgap, which may be caused by the space hindrance effect. The dynamic switching abilities of the polymers were obtained by the chronoabsorptometry method, and the results also suggested that the alkoxy thiophene-containing polymers (as π-bridges) have higher contrast ratios than the corresponding alkyl thiophene-containing polymers. Furthermore, the increase in the length of the side alkyl chain might have a detrimental effect on the optical contrast ratios of the polymers.

## 1. Introduction

Conjugated polymers (CPs) could be considered as a type of organic semiconductor material, which generally have expected electrical and optical properties based on the band gap theory [1]. Fundamentally, the band gap is defined as the difference in the energy gap between the valence band and the conduction band of CPs, which is also the energy difference between the highest occupied molecular orbital (HOMO) and lowest unoccupied molecular orbital (LUMO) [2]. It is considered that bandgap values are the results of many factors, including planarity, resonance effects, interchain effects, and bond length alternation [3]. The alternating distribution of one donor unit and one acceptor unit along a polymer backbone is the basic feature of the donor-acceptor (D-A) theory, which is considered to be the most effective way to reduce the bandgaps of polymers, by promoting intramolecular charge transfers and then improving the resonance effects on the polymer backbone [4,5]. The D-A approach has been widely exploited for the preparation of desirable CPs for industrial or academic applications such as those of electroluminescence devices [6], polymer solar cells [7,8], and memory devices [9], as well as energy storage devices [10]. Besides, a lot of applications of CPs are based on their electrochromic properties, such as energy-saving intelligent windows, nonemissive display devices, glareproof mirrors, and camouflage materials [11]. The absorption bands (colors) of the films of electrochromic CPs reversibly change during the redox process of the polymers, driven by the external voltage applied. Compared with inorganic electrochromic compounds, such as WO_3_ and IrO_2_, soluble polymeric electrochromic materials have some merits, including the ease of the fabrication of large films, bright colors, large optical contrast ratios, and fast response speeds [12]. Furthermore, the bandgaps of the D-A-type polymers can be easily modulated by structural modification through the selection of proper donor or acceptor units for stronger or weaker ones, or by changing the donor-to-acceptor ratios within the polymer backbone [13].

Poly(thiophenes) represent an important class of electrochromic materials due to their intense color changes, robust environmental and redox stabilities, and facile structural modifications. Soluble poly(thiophenes) have been prepared by substituting the 3- or 4-, or both 3- and 4- positions of the thiophene ring with flexible units, such as alkyl, alkoxy, or alkylenedioxy units [12]. Thiophenes and their derivatives are also frequently used as the donor units for the preparation of D-A-type polymers with the acceptor units (electron-deficient units) [12]. In addition, as redox-active chromophores, the alkyl-substituted carbazole and fluorene has been used as the donor units for the construction of D-A-type polymeric electrochromic materials, which usually present multichromic properties, depending on the selection of the constitutional units [14,15,16].

The replacement of sulfur atoms with selenium in the π-conjugated polymers represents another method whereby to regulate and adjust the structure of the resultant polymers, which usually leads to the reduction in bandgap and the bathochromic shift in absorption spectra of the polymers, due to the larger size and reduced electronegativity of the Se atom compared to that of the S atom. Meanwhile, the formation of interchain Se–Se interactions could also be anticipated and be attributed to the better polarizability of the Se atom compared to the sulfur atom, which leads to the higher charge carrier mobility of the Se-containing polymers [17,18,19]. Selenophene and benzoselenadiazole (BSe) are two common heteroaromatic compounds and carriers of Se atoms, which are used as electron-donating and electron-deficient building blocks, respectively, for the preparation of D-A-type conjugated polymers [17,18,19]. 

Besides the BSe unit, its derivatives including 4,7-dibromo-5,6-bis(octyloxy)-[2,1,3]-benzoselenadiazole (BSeoc) or 4,7-bis(5-bromothiophene-2-yl)-5,6-bis(octyloxy)-[2,1,3]-benzoselenadiazole (DTBSe) have also been employed as electron-accepting units [17], and the introduction of the alkoxy units was considered to be able to increase the solubility of the resultant polymers. Some donor units have been chosen as the counter monomers for the synthesis of D-A-type polymers with BSe or BSe derivatives, including alkyl-substituted carbazole (Cz) [20], cyclopentadithiophene [19], fluorene [21], benzo[1,2-*b*:4,5-*b*′]dithiophene (BDT) [22], dialkoxy-*p*-phenylene (Ph) [23], and so forth. Hyun reported that the replacement of thiophene with selenophene in the BSeoc-containing polymers led to the lower bandgap and higher photovoltaic performance compared to its thiophene-containing analogues [8]. The copolymer incorporating 9,9-dioctylfluorenyl and benzoselenadiazole has been used as a fluorescent probe for cellular imaging, and an optical quantum yield of 44% was obtained from the copolymer dots at a fluorene-to-benzoselenadiazole ratio of 70:30 [21]. BSe- or selenophene-containing conjugated polymers were also prepared and characterized in terms of their electrochromic properties, which usually showed lower bandgaps and higher redox stabilities than that of their analogues [4,24]. A soluble copolymer consisting of BSe and 2,5-bis-dithienyl-1*H*-pyrrole has been synthesized by Koyuncu et al.; the polymer showed high optical contrast, fast doping and dedoping speed, and robust stabilities during the redox process [4]. To date, the reports on soluble BSe-containing electrochromic polymers are few, which should be further extended and strengthened due to the good application records of BSe and its derivatives in the field of organic photovoltaics (OPVs) [25].

**Scheme 1:**
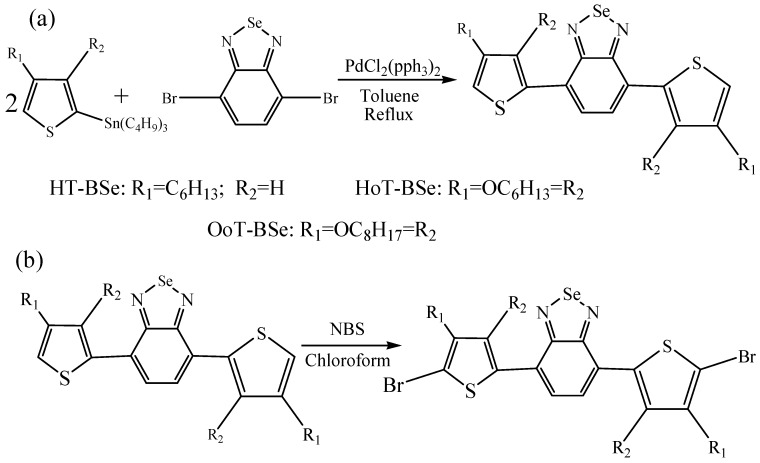
Synthesis and chemical structures of BSe-containing derivatives. (**a**) The synthesis of HT-BSe, HoT-BSe and OoT-BSe; (**b**) The bromination reaction of the above monomers.

**Scheme 2:**
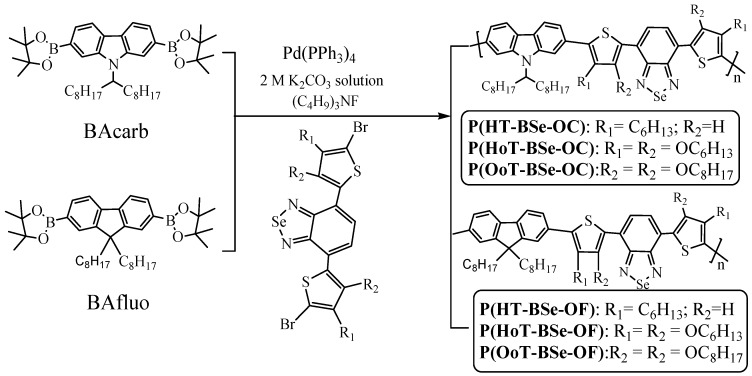
Synthesis and chemical structures of the polymers.

In this report, we prepared six conjugated polymers with 4,7-bis(5-bromo-4-hexythiophen-2-yl)benzo[c][1,2,5]selenadiazole (2Br-HT-BSe), 4,7-bis(5-bromo-3,4-bis(hexyloxy)thiophene-2-yl)benzo[c][1,2,5]selenadiazole(2Br-HoT-BSe), and 4,7-bis(5-bromo-3,4-bis(octyloxy)thiophene-2-yl)benzo[c][1,2,5]selenadiazole (2Br-OoT-BSe) as the electron accepting units, and 9-(octylnonyl-9-yl)-2,7-bis(4,4,5,5-tetramethyl-1,3,2-dioxaborolan-2-yl)-9*H*-carbazole (BAcarb) or 2,7-bis(4,4,5,5-tetramethyl-1,3,2-dioxaborolan-2-yl)-9,9-dioctylfluorene (BAfluo) as the electron-donating units. The structures of the above three acceptor units and six alternating copolymers are shown in Scheme 1 and Scheme 2. The effects of the side-chain substituent (located on the thiophene ring) and the electron-donating units on the electrochromic properties of the resultant polymers are summarized and discussed in detail.

## 2. Experimental Section

### 2.1. Materials

4,7-dibromo-2,1,3-benzoselenadiazole (BSe), 3-hexylthiophene, 3,4-dihexyloxythiophene, and 3,4-dioctyloxythiophene were all purchased from Puyang Huicheng Electronic Material Co., Ltd., (Puyang, China) and were used as received. The 2-substituted tributylstannane derivatives of the thiophene derivatives were prepared with the methods reported previously [25]. Chlorotributyltin, *n*-butylithium, acetonitrile (ACN), chloroform, toluene, *N*-bromosuccinimide (NBS), bis-(triphenylphosphine)dichloropalladium (Pd(PPh_3_)_2_Cl_2_), tetrakis(triphenyphosphine)palladium (Pd(PPh_3_)_4_), tetrabutylammonium hexafluorophosphate (TBAPF_6_, 98%), methanol, and acetone were all obtained from Aladdin Chemical Co., Ltd., Shanghai, China and used as received. Indium-tin-oxide-coated (ITO) glass (sheet resistance: <10 Ω/sq, purchased from Shenzhen CSG Display Technologies, Shenzhen, China) was cleaned thoroughly before use. 9-(1-octylnonyl-9-yl)-2,7-bis(4,4,5,5-tetramethyl-1,3,2-dioxaborolan-2-yl)-9*H*-carbazole (BAcarb) and 2,7-bis(4,4,5,5-tetramethyl-1,3,2-dioxaborolane-2-yl)-9,9-dioctylfluorene (BAfluo) were purchased from Derthon Optoelectronic Materials Sci. Tech. Co. Ltd. (Shenzhen, China ) and used without further purification.

### 2.2. Instrumentation and Characterization

^1^H NMR and ^13^C NMR spectra were obtained by a Varian AMX 400 spectrometer (Varian Inc., Santa Clara, CA, USA) at 400 MHz with tetramethylsilane as the internal standard substance. Molecular weights (number-average, *M*_n_ and weight-average, *M*_w_) and polydispersity indices (PDI) were performed through gel permeation chromatography (GPC) (Viscotek module-350 system, Viscotek, Houston, TX, USA), chloroform was used as the solvent (2 mg/mL) at a flow rate of 1 mL/min at 40 °C, and calibrated polystyrene was used as the standard. Thermogravimetric analysis (TGA) measurements were conducted on a NETZSCH STA 499C TG-DSC apparatus (Netzsch Inc., Bavaria, Germany) under a nitrogen atmosphere at a heating rate of 15 K·min^−1^. The cyclic voltammetry (CV) of the polymer was performed by using an Autolab potentiostat/galvanostat (PGSTAT 302N, Metrohm Autolab, Utrecht, The Netherlands), employing a glassy carbon electrode (5 mm diameter, 0.196 cm^2^) coated with the polymer films as the working electrode, a platinum wire as the counter electrode, and a Ag wire (0.02 V vs. SCE) as the pseudo-reference electrode. The supporting electrolyte used for all electrochemical measurements was 0.1 M TBAPF_6_ in ACN solution. UV-Vis-NIR (near-infrared region) absorption spectra were recorded on a Varian Cary 5000 spectrophotometer (Varian Inc., Santa Clara, CA, USA). The spectroelectrochemistry and chronoabsorptometry was conducted by the joint unit of the spectrophotometer and the potentiostat/galvanostat, with the former used to record the change in spectral absorption change, and the latter used to control the potentials biased on the polymer-coated ITO electrode. The polymer films were prepared by spraying the polymer solution (4 mg/mL) on the ITO plate (0.9 cm × 1.8 cm), with the thickness of the polymers controlled with the absorbance value. The three-electrode system was constructed on a quartz cell, with the polymer-coated ITO electrode as the working electrode (WE) electrode, and the Pt and Ag wires were used as the counter and reference electrodes, respectively. The chromatic coordinates of the polymers under different potentials were measured with the same apparatus as used in the spectroelectrochemical measurement with the employment of the chromaticity software (Varian Inc., Santa Clara, CA, USA). The standard illuminant D65 with a 2° observer at constant temperature in a light booth designed to exclude external light was used. Prior to each set of measurements, background color coordinates (*x*, *y*, and *z* values) were taken at open-circuit, using the electrolyte solution without the polymer films under study. Digital photographs of the polymer films were taken by a Canon (Canon Inc., Tokyo, Japan) Power Shot A3000 IS digital camera.

### 2.3. Synthesis of the 2,7-Substituted BSe with Thiophene Derivatives

#### 2.3.1. Synthesis of HT-BSe

A mixture solution consisting of 4 mmoL of BSe, 12 mmoL of tributyl(4-hexylthiophen-2-yl)stannane, 60 mL of toluene, and 0.36 mmoL of Pd(PPh_3_)_2_Cl_2_ was mixed in a round-bottom flask (Scheme 1). Then, the flask was immersed in an oil bath with electromagnetic stirring at 100 °C, and the mixture solution was isolated from the air by maintaining a positive pressure with a balloon filled with argon gas. Before the flask was filled with argon gas, the air in the flask was pumped out by a vacuum pump. The reaction was terminated after 24 h, and then the solvent was distilled off under vacuum conditions [17]. The raw product in the residue was purified by a silica gel column chromatography method with a yield of 65% (1:2 DCM: *n*-hexane). ^1^H NMR (CDCl_3_, 400 MHz, ppm): δ = 7.87 (d, 2H, ArH), 7.75 (s, 2H), 7.05 (d, 2H), 2.68 (m, 4H), 1.69 (m, 4H), 1.33 (m, 12H), 0.91 (m, 6H). 13C NMR (CDCl_3_, δ, ppm): δ = 158.16, 144.00, 139.30, 128.87, 127.38, 125.75, 121.86, 31.75, 30.66, 30.50, 29.11, 22.69, 14.18 (see Supporting Information, Figure S1).

#### 2.3.2. The Synthesis of HoT-BSe

The same procedure was used to prepare HoT-BSe with a yield of 64.5%. ^1^H NMR (CDCl_3_, 400 MHz, ppm): δ = 8.28 (s, 2H, ArH), 6.37 (s, 2H), 4.08 (t, 4H), 4.01(t, 4H), 1.83 (m, 4H), 1.69 (m, 4H), 1.36 (m, 16H), 1.23 (m, 8H), 0.92 (m, 6H), 0.83 (m, 6H). ^13^C NMR (CDCl_3_, δ, ppm): δ = 158.47, 150.16, 144.95, 128.17, 125.96, 120.96, 98.38, 72.91, 69.98, 31.56, 30.10, 29.18, 25.84, 25.66, 22.64, 14.08 (see Figure S2).

#### 2.3.3. The Synthesis of OoT-BSe

The product was obtained as a reddish-brown color with a yield of 62.5%. ^1^H NMR (CDCl_3_, 400 MHz, ppm): δ = 8.24 (s, 2H, ArH), 6.37 (s, 2H), 3.91 (m, 8H), 1.31 (m, 48H), 0.81 (m, 12H). ^13^C NMR (CDCl_3_, δ, ppm): δ = 158.45, 150.30, 145.34, 132.32, 125.96, 120.71, 114.45, 98.70, 75.09, 72.21, 40.17, 39.43, 30.58, 29.06, 28.21, 26.71, 23.64, 22.99, 17.21, 14.04, 14.00, 13.53, 11.13 (see Figure S3).

#### 2.3.4. General Procedure for the Bromination Reaction of HT-BSe, HoT-BSe, and OoT-BSe (Scheme 1)

In a single-neck round-bottom flask (1 L), 3 mM of HT-BSe was dissolved in a mixture of chloroform (500 mL) and glacial acetic acid (100 mL), and the flask was then equipped with a drying tube. 7.2 mM of NBS was added to the above solution in four batches, with a time interval of 5 h. The reaction proceeded at room temperature overnight, after which the above mixture was poured into 500 mL of a saturated solution of Na_2_SO_3_ and extracted with dichloromethane twice. The organic layer was combined together and dried with anhydrous magnesium sulfate. After removing magnesium sulfate by filtration, the solvent was then removed under reduced pressure. The crude product was purified by column chromatography with n-hexane and dichloromethane (1:1 volume ratio) as the eluent, and 2Br-HT-BSe was obtained as a deep-red solid with a yield of 89% [26]. ^1^H NMR (CDCl_3_, 400 MHz, ppm): δ = 7.52 (d, 2H, ArH), 7.44 (s, 2H), 2.57 (t, 4H), 1.62 (m, 4H), 1.33 (m, 12H), 0.90 (t, 6H). ^13^C NMR (CDCl_3_, δ, ppm): δ = 161.97, 147.09, 143.22, 131.98, 130.89, 129.11, 116.87, 36.32, 34.43, 34.27, 33.69, 27.32, 18.83 (see Figure S4).

##### The Synthesis of 2Br-HoT-BSe

The procedure was the same as that of the HT-BSe. 2Br-HoT-BSe was also obtained as a reddish-brown color with a yield of 89%. ^1^H NMR (CDCl_3_, 400 MHz, ppm): δ = 8.26 (d, 2H, ArH), 4.13 (t, 4H), 4.06 (t, 4H), 1.75 (m, 8H), 1.50 (m, 4H), 1.35 (m, 12H), 1.27 (m, 8H), 0.91 (t, 6H), 0.85 (t, 6H). ^13^C NMR (CDCl_3_, δ, ppm): δ = 157.55, 147.90, 147.32, 127.09, 124.95, 121.35, 100.99, 73.74, 31.53, 30.02, 25.60, 25.55, 22.50, 14.15 (see Figure S5).

##### The Synthesis of 2Br-OoT-BSe

The product was also a reddish-brown color, and the yield was 87%. ^1^H NMR (CDCl_3_, 400 MHz, ppm): δ = 8.27 (d, 2H, ArH), 4.04 (t, 4H), 3.95 (t, 4H), 1.44 (m, 24H), 1.23 (m, 10H), 0.95 (m, 14H), 0.84 (t, 12H). ^13^C NMR (CDCl_3_, δ, ppm): δ = 157.89, 148.30, 147.83, 127.64, 125.21, 121.16, 100.55, 76.71, 76.50, 76.21, 40.27, 40.23, 30.27, 30.21, 29.12, 29.07, 23.58, 23.13, 23.07, 14.17, 14.07, 11.17, 11.04 (see Figure S6).

#### 2.3.5. Copolymer Syntheses

##### General Procedure for the Synthesis of the Copolymers (Scheme 2)

**Figure 1:**
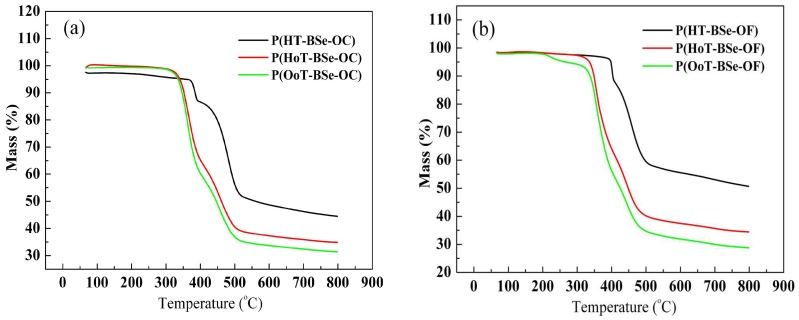
Thermogravimetric analysis (TGA) curves of (**a**) carbazole-based copolymers; (**b**) fluorene-based copolymers.

The synthetic route and the chemical structure of the new polymers, namely P(HT-BSe-OC), P(HoT-BSe-OC), P(OoT-BSe-OC), P(HT-BSe-OF), P(HoT-BSe-OF), and P(OoT-BSe-OF), are depicted in Scheme 2 and Figure 1, respectively. All of the copolymers were synthesized using the Suzuki-Miyaura cross-coupling reaction, and the typical procedure was described as follows, taking the synthesis of P(HT-Bse-OC) as an example [2,8,20]. 1 mmol of 2Br-HT-Bse, 1 mmol of BAcarb, and 0.03 mmol of Pd(PPh_3_)_4_ were dissolved in 60 mL of the mixture of toluene and 2 M K_2_CO_3_ solution (3:2 *v*/*v*) under nitrogen gas protection. Then, 0.01 mmol of tetrabutylammonium fluoride (TBAF) was added to the above mixture. The flask containing the above mixture was heated to 90 °C under an argon atmosphere and stirred magnetically. The reaction proceeded for 48 h, and then the mixture was cooled down to room temperature. The solvent was removed under vacuum pressure, and the residue was dissolved in 100 mL of chloroform, which was rinsed with excess water to remove K_2_CO_3_ from the mixture. The organic phase was extracted with chloroform and was combined together after two rinsing processes. After being dried with anhydrous magnesium sulfate, the organic phase was concentrated, and the precipitation solid was obtained by adding methanol to the concentrated solution. The crude product was collected and then refluxed in a Soxhlet apparatus using methanol and acetone, respectively, for 24 h to remove oligomers and catalyst residues, and the bright purple polymer P(HT-BSe-OC) was obtained finally with a yield of 78%.

^1^H NMR (CDCl_3_, 400 MHz, ppm): δ = 8.17 (s, 2H), 8.03 (s, 2H), 7.85 (s, 2H), 7.74 (s, 1H), 7.57 (s, 1H), 7.45 (s, 2H), 4.63 (s, 1H), 2.87 (s, 4H), 2.36 (s, 2H), 1.98 (s, 2H), 1.78 (s, 4H), 1.49–0.97 (d, 36H), 0.96–0.66 (d, 12H) (see Figure S7).

##### Synthesis of P(HoT-BSe-OC)

The same synthetic procedure was employed for the preparation of P(HoT-BSe-OC), with a yield of 77%. P(HoT-BSe-OC) was obtained as a violet-blue powder. ^1^H NMR (CDCl_3_, 400 MHz, ppm): δ = 8.40 (s, 2H), 8.13 (d, 3H), 7.98 (s, 1H), 7.68 (s, 2H), 4.16 (d, 9H), 2.36 (s, 2H), 2.02 (s, 2H), 1.83 (d, 8H), 1.47 (s, 8H), 1.38–1.07 (d, 40H), 1.73–1.01 (m, 18H) (see Figure S8).

##### Synthesis of P(OoT-Bse-OC)

The polymer P(OoT-Bse-OC) was obtained as a hyacinth-violet color with a yield of 81%. ^1^H NMR (CDCl_3_, 400 MHz, ppm): δ = 8.38 (s, 2H), 8.14 (s, 2H), 8.02 (s, 1H), 7.91 (s, 1H), 7.73 (d, 2H), 4.07 (d, 8H), 3.77 (d, 1H), 2.42 (s, 1H), 2.05 (s, 1H), 1.79 (s, 3H), 1.53–1.05 (m, 56H), 0.98–0.77 (m, 32H) (see Figure S9).

##### Synthesis of P(HT-BSe-OF)

The polymer P(HT-BSe-OF) was a red-lilac powder, and the yield was 82%. ^1^H NMR (CDCl_3_, 400 MHz, ppm): δ = 7.98 (s, 2H), 7.88–7.74 (m, 3H), 7.52 (s, 3H), 5.30 (s, 2H), 2.81 (s, 2H), 2.04 (s, 1H), 1.75 (s, 2H), 1.42–1.02 (m, 36H), 0.91–0.59 (m, 21H) (see Figure S10).

##### Synthesis of P(HoT-BSe-OF)

The polymer P(HoT-BSe-OF) was a lilac-purple powder, and the yield was 80%. P(HoT-BSe-OF): ^1^H NMR (CDCl_3_, 400 MHz, ppm): δ = 8.40 (s, 2H), 8.01–7.62 (m, 6H), 4.14 (d, 8H), 2.07 (s, 4H), 1.89–1.68 (m, 8H), 1.48 (s, 8H), 1.40–1.23 (m, 17H), 1.15 (d, 21H), 0.99–0.61 (m, 22H) (see Figure S11).

##### Synthesis of P(OoT-BSe-OF)

The color of P(OoT-BSe-OF) powder was lilac-purple and the yield of the polymer was 85%. ^1^H NMR (CDCl_3_, 400 MHz, ppm): δ = 8.32 (s, 2H), 7.79 (s, 4H), 7.73 (s, 2H), 4.02 (d, 8H), 2.06 (s, 3H), 1.75 (s, 3H), 1.28 (t, 53H), 0.98–0.73 (m, 34H), 0.74–0.60 (m, 3H) (see Figure S12).

## 3. Results and Discussion

### 3.1. Molecular Weight and Thermogravimetric Analysis

The molecular weight and polydispersity of polymers are the important parameters for electrochromic applications. A high molecular weight favors the performance of electrochromic devices in several aspects, including the improvement of the adhesion to the substrate, the resistance to dissolving in the supporting electrolyte, and the maintenance of a high optical contrast after repeated electrochromic switches [27,28,29]. Table 1 presents the synthetic yield, the weight-average molecular weight (*M*_w_), the number-average molecular weight (*M*_n_), the polydispersity, and the thermal data of six polymers. The molecular weights of the polymers against polystyrene standards were determined by gel permeation chromatography (GPC). The GPC results showed that the *M*_w_ weights of the polymers obtained were in the range of 14.3–16.9 kDa with polydispersity (*M*_w_/*M*_n_) of 2.0–2.4. Higher *M*_n_ and *M*_w_ values were found for the HT-BSe-containing polymers compared to the other types of polymers, which might be attributed to the smaller steric hindrance effect of the HT-BSe, since it only has two symmetrical hexyl side chains on the thiophene ring. The thermal stabilities of the polymers are analyzed by the TGA method. As shown in Table 2, the thermal decomposition temperatures (*T*_d_) were higher than 310 °C, and *T*_d_ is defined as the temperature at which 5% weight loss of the original mass happens. It was found that the HT-BSe-containing polymers had higher *T*_d_ values than that of the HoT-BSe- or OoT-BSe-containing polymers, with the latter kinds of polymers having similar *T*_d_ values (Figure 1). This suggested that the thermal stabilities of the HT-BSe-containing polymers are superior to those of the HoT-BSe- or OoT-BSe-containing polymers. Furthermore, the selection of carbazole or fluorene as the donor unit had a very limited effect on the thermal stability of the resultant polymers. The thermal stability of the polymers could meet the requirements of the operating conditions of electrochromic devices, in most cases. All polymers had good solubility in chloroform (≥4 mg/mL), but had low solubility in other solvents including tetrahydrofuran, toluene, o-xylene, and so forth.

### 3.2. Electrochemical Properties

**Figure 2:**
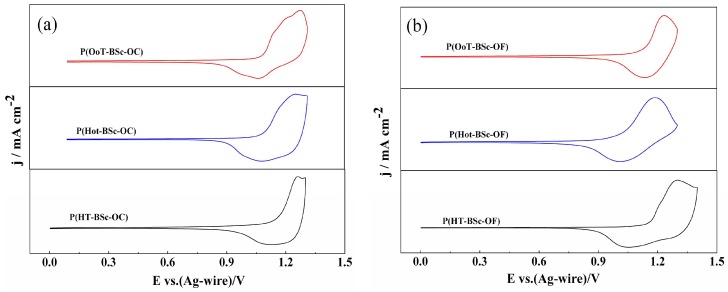
Cyclic voltammograms of (**a**) carbazole-based copolymers; (**b**) fluorene-based copolymers.

For electrochemical characterization, the polymers were dissolved in chloroform to a concentration of 4 mg/mL and then sprayed onto a glassy carbon electrode. Upon solvent evaporation, the polymer films were subjected to cyclic voltammetry (CV) studies under ambient conditions (See Experimental Section). As shown in Figure 2, well-defined redox couples were found for all of the polymers, with the redox peaks located at 1.25 V/1.12 V for HT-BSe-OC, 1.23 V/1.04 V for P(HoT-BSe-OC), 1.26 V/1.03 V for P(OoT-BSe-OC), 1.30 V/1.03 V for P(HT-BSe-OF), 1.20 V/1.00 V for P(HoT-BSe-OF), and 1.23 V/1.13 V for P(OoT-BSe-OF). The initial oxidation potentials (*E*_onset_) of the polymers are also displayed in Table 2. The polymers containing HoT-BSe units had lower *E*_onset_ potentials compared to the polymers containing HT-BSe units, due to the stronger electron-donating abilities of 3,4-dihexyloxythiphene compared to 3-hexylthiophene, which improved the conjugation effects of the polymers comprising P(HoT-BSe-OC) and P(HoT-BSe-OF) [30,31]. On the other hand, the acyclic side chains of the dioxythiophenes cause steric repulsions along the polymer backbone that decrease the effective conjugation length of the copolymers, which could offset the stronger electron-donating ability of the 3,4-dihexyloxythiphene unit to some extent [10]. Thus, only slight decreases in the *E*_onset_ potentials were observed for the 3,4-dihexyloxythiphene-containing polymers (P(HoT-BSe-OC) and P(HoT-BSe-OF)) compared to those of the 3-hexylthiphene-containing polymers (P(HoT-BSe-OC) and P(OoT-BSe-OF)), in spite of the presence of two alkoxy units with strong electron-donating abilities on the 3,4 sites of the thiophene unit in the former kind of polymers. Because the 3,4-dioctoxythiophene units have longer side chains than those of the 3,4-dihexyloxythiophene units, the former has a larger steric effect than that of the latter unit. Thus, the *E*_onset_ potentials of the 3,4-dioctoxythiophene-containing polymers (P(OoT-BSe-OC) and P(OoT-BSe-OF)) were larger than those of the 3,4-dihexyloxythiophene-containing polymers (P(HoT-BSe-OC) and P(HoT-BSe-OF)) (Table 2).

### 3.3. Optical Properties

**Figure 3:**
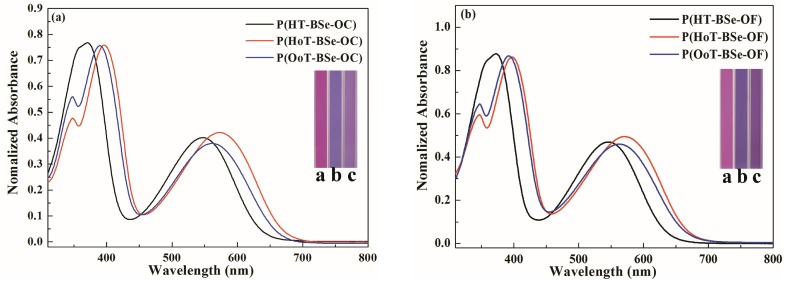
Normalized UV-Vis spectra of polymer solutions and their photographs (inset pictures). (**a**) Carbazole-based copolymers; (**b**) fluorene-based copolymers.

The UV-Vis spectra of the polymer solutions are shown in Figure 3, with the inset photographs of the solutions in the cuvettes taken under white light. In the chloroform solution, the spectra of all six polymers displayed two distinct absorption bands, which were characteristic of polymers incorporating alternating donor–acceptor units along the polymer backbone [32]. The low-energy absorption peak usually arises from the π–π* transition along the D-A-type polymer backbone.

**Figure 4:**
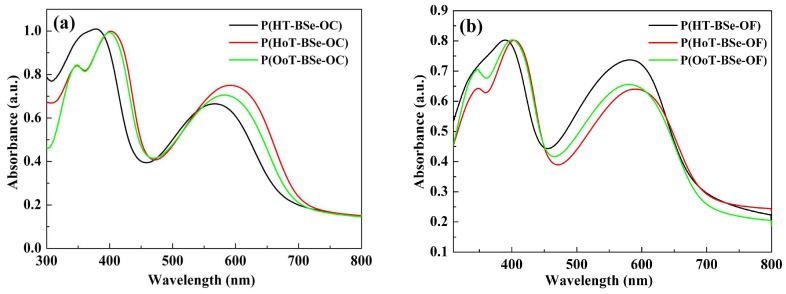
Normalized UV-Vis spectra of polymer films with no externally added potentials: (**a**) three carbazole-based copolymers; (**b**) three fluorene-based copolymers.

The absorption peaks of the polymers in both solution and solid film states are listed in Table 2. The λ_max_ refers to the wavelength of the absorption peaks, and the λ_onset_ is defined as the starting edge of the low-energy absorption, which is obtained from the intersection of the baseline and the tangent line of the second π–π* transition. Considering the λ_max_ of the low-energy absorption peak of the six polymers in solution (from the shortest to longest), the order is as follows: P(HT-Bse-OF) (545 nm), P(HT-Bse-OC) (547 nm), P(OoT-Bse-OC) (561 nm), P(OoT-Bse-OF) (562 nm), P(HoT-Bse-OF) (569 nm), and P(HOT-Bse-OC) (572 nm). The bathochromic shift of the absorption maximum of the 3,4-dialkoxythiophene-containing polymers to that of the 3-hexylthiophene-containing polymers is as expected, because the 3,4-dialkoxythiophene moiety has a stronger electron-donating ability than that of 3-hexylthiophene, which leads to an enhanced intramolecular charge transfer (ICT) along the polymer backbone. In addition, the growth of the alkyl chains on the 3,4-dialkoxythiophenes could induce stronger steric repulsions along the polymer backbone, which could decrease the effective conjugation length and the blue shift of the absorption peaks, which would explains the blue-shift of the absorption bands of the 3,4-dioctyloxythiophene-containing polymers compared with that of the 3,4-dihexyloxythiophene-containing polymers [5,33]. The substitution of carbazole or fluorene in the polymer backbone does not cause a significant change in the positions of the absorption peaks of the resultant polymers, which might be due to their similar structure and electron-donating abilities as electron donor units within the polymers (Table 2). The colors of the carbazole-containing polymers in solution are shown in the inset of Figure 4a, and exhibit dark magenta, dark slate-blue, and dark violet colors for P(HT-BSe-OC), P(OoT-BSe-OC), and P(HoT-BSe-OC), respectively. The colors of the corresponding fluorene-containing polymers are also shown in Figure 3a; it is apparent that the polymers containing the same BSe-based acceptor units (e.g., HT-BSe) showed almost the same colors.

The spectra of the neutral state films prepared by the spray method on the ITO electrode were obtained, as shown in Figure 4. The data of the λ_max_ and the λ_onset_ values are also shown in Table 2, which show a similar change rule to that of the data from the spectra of the polymer solutions. Specifically, the bandgap values of the polymers in the solid state were calculated from the well-established equation: *E*_g_ (eV) = 1241/λ_onset_ (nm). The bandgaps of the six polymers in order from high to low are: P(HT-BSe-OC) (1.78 eV), P(HT-BSe-OF) (1.75 eV), P(OoT-BSe-OC) (1.74 eV), P(OoT-BSe-OF) (1.74 eV), P(HoT-BSe-OF) (1.72 eV), and P(HoT-BSe-OC) (1.71 eV). It was observed that there is a slight decrease in the bandgaps of the 3,4-dialkoxythiophene-containing polymers compared to those of the 3-hexylthiophene-containing polymers. The strong electron-donating ability of 3,4-dialkoxythiophenes has been offset by its steric repulsions along the polymer backbone, which could account for the slight difference between the bandgaps of the 3,4-dialkoxythiophene-containing polymers and the 3-hexylthiophene-containing polymers. There were no obvious changes in the shape and relative displacement of the absorption peaks of the polymer films relative to the ones in the solution state. Besides, there were red-shifts in the absorption curves of the polymer films relative to their corresponding absorption curves in solution. The presence of the π–π stacking of the polymer chains in the solid state might account for the red-shifted λ_max_ [34].

### 3.4. Spectroelectrochemistry

**Figure 5:**
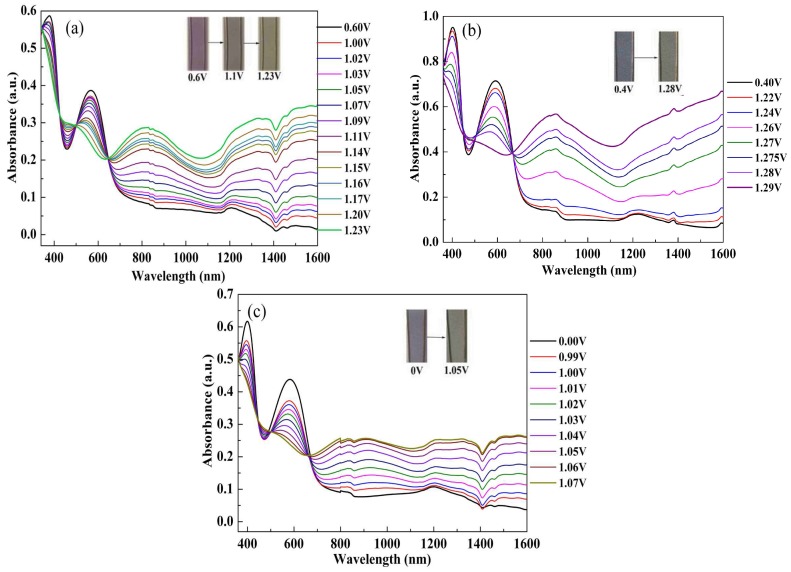
Spectroelectrochemical spectra of three carbazole-based copolymers. (**a**) P(HT-BSe-OC); (**b**) P(HoT-BSe-OC); (**c**) P(OoT-BSe-OC).

The UV-Vis-NIR spectra were monitored between the dedoped and doped states in order to explain the electro-optical characteristics of the polymer films. The polymer-coated ITO glass was used as the working electrode, which was obtained by a spraying process from a solution of 4 mg/mL polymer in chloroform. After drying under ambient temperatures, the films were subjected to subsequent redox cycling by CV to achieve redox stability prior to spectroelectrochemical measurement. The spectroelectrochemical spectra of P(HT-BSe-OC), P(HoT-BSe-OC), and P(OoT-BSe-OC) are shown in Figure 5, and the photographs of the neutral, intermediate, and fully oxidized states of the thin polymer films are illustrated in the insets of the figures. The absorption spectra of the polymers are similar to each other in the dedoped state, both having two π–π* transition bands. The changes in the absorption spectra were recorded when the potentials were gradually increased from 0.60 to 1.23 V for P(HT-BSe-OC), 0.40 to 1.29 V for P(HoT-BSe-OC), and 0.0 to 1.07 V for P(OoT-BSe-OC). The high-energy absorptions of the polymers are located at 377, 402, and 399 nm, respectively, for the abovementioned three polymers, which were mainly centered in the UV region, thus giving little contribution to the color perceived by the human eye.

The polymer films were gradually oxidized from their neutral forms to the doped states in determined potential steps. During these processes, the changes of the absorption spectra of the six polymers follow a similar tendency. As shown in Figure 5a, for P(HT-BSe-OC), both of the π–π* transition bands gradually decreased and two new absorption bands were simultaneously produced, with one centered at 829 nm and the other extended to the infrared region (higher than 1358 nm) due to the production of polaron and bipolaron bands, respectively. At the neutral state, P(HT-BSe-OC) has a purply color (+0.6 V), then changes to a dark brown color at an intermediate potential (+1.1 V), and finally to a transparent light-gray color at the fully oxidized state (Figure 5a). Upon oxidation, the P(HoT-BSe-OC) film showed a color switch from blue-black color to light-gray color, and its polaron and bipolaron bands appeared at 855 nm and beyond 1200 nm, respectively (Figure 5b). For the P(OoT-BSe-OC) film, the color changed from blue-purple (0 V) to gray color (+1.05 V) when it was oxidized gradually (Figure 5c). Similarly, the wavelength of the peaks for the occurrence of polaron and bipolaron were 904 nm and beyond 1200 nm, respectively, for P(OoT-BSe-OC). All three polymers could be reversibly interconverted between the reduced and the oxidized states, as indicated by the presence of the isosbestic points at 643, 667, and 667 nm for P(HT-BSe-OC), P(HoT-BSe-OC), and P(OoT-BSe-OC), respectively.

The spectroelectrochemistry of three fluorene-containing polymers were also conducted and the data are presented in Figure S13 (see Supporting Information). The spectroelectrochemical spectra of the fluorene-containing polymers had almost the same change patterns of the carbazole-based copolymers containing the same acceptor units. With the transition from the reduced to the oxidized states, the charged transitional states, including the polaron and bipolaron, also appeared on the fluorene-containing polymers. Furthermore, the color switches of the fluorene-containing copolymers are similar to those of their corresponding carbazole-based copolymers.

### 3.5. Chronoabsorptometry

**Figure 6:**
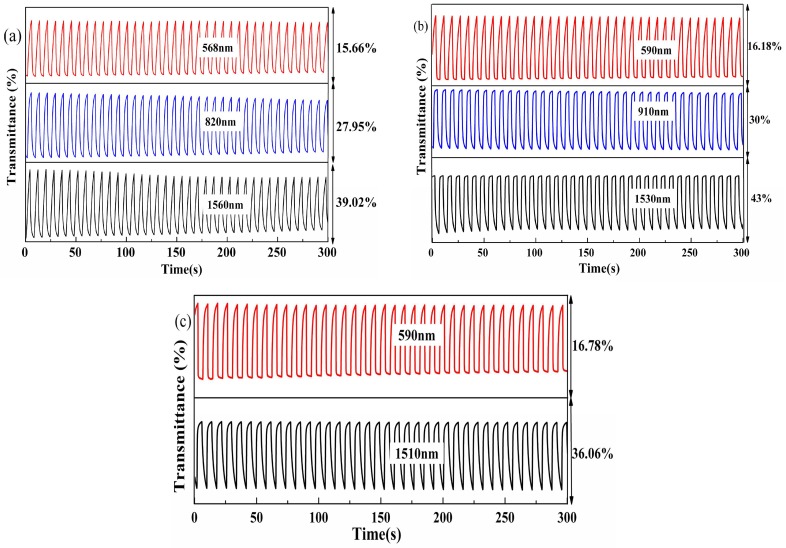
The chronoabsorptometry of three carbazole-based polymers with the interval time of 5 s in the square wave potential method. The test wavelengths and the corresponding contrast ratios are labeled in the figures. (**a**) P(HT-BSe-OC); (**b**) P(HoT-BSe-OC); (**c**) P(OoT-BSe-OC).

**Figure 7:**
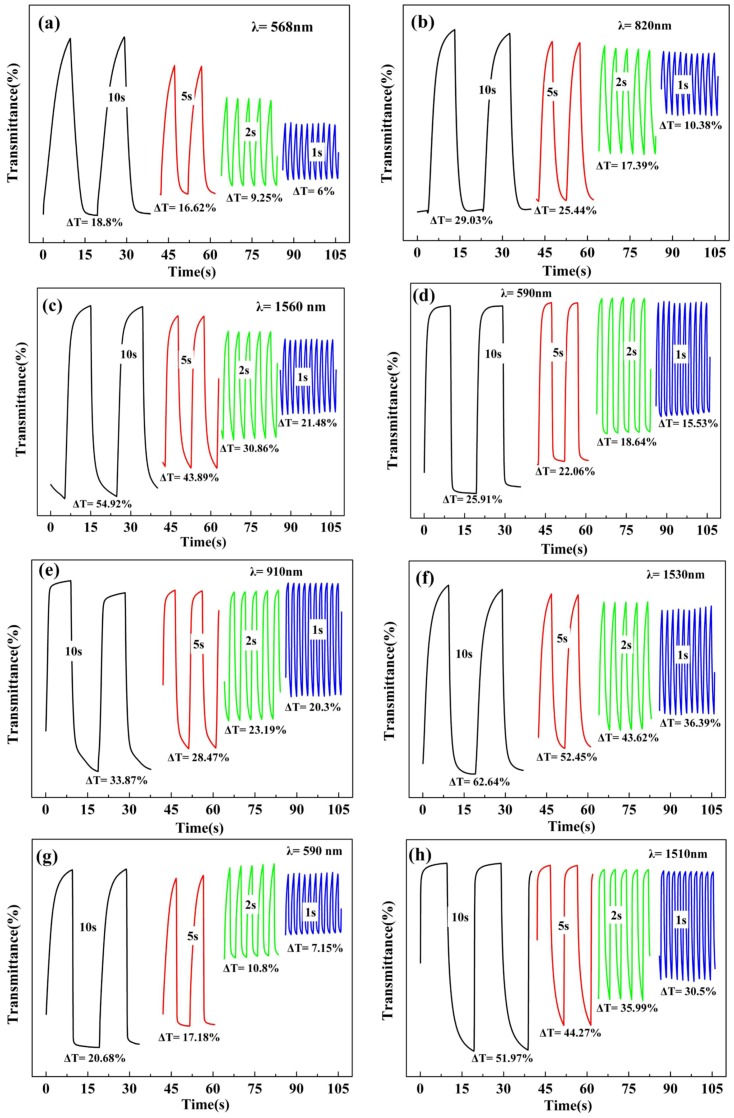
The dependence of the optical contrast ratios (∆*T*) on the interval time in the chronoabsorptometry study. The interval times tested in the in the square wave potential method were varied at 10, 5, 2 and 1 s in turn. The test wavelengths and the corresponding contrast ratios are labeled in the figures. (**a**–**c**), P(HT-BSe-OC); (**d**–**f**): P(HoT-BSe-OC); (**g**, **h**): P(OoT-BSe-OC).

For a comprehensive understanding of the electrochromic properties of the polymers, it is necessary to study the optical contrasts and corresponding response times during the dynamic conversion of the polymer between the neutral and oxidized states. The polymer-coated ITO/glass electrode was used as the working electrode, and Pt wire and Ag wire were used as the counter electrode and reference electrode, respectively. The electrode system was reconstructed in the quartz cuvette, and the working electrodes were immersed in the 0.1 M TBAPF_6_ ACN solution, and then stabilized with 10 rounds of cyclic voltammetry before measurement. The polymer films were switched between the neutral and the oxidized states using potential square waves in intervals of 1–10 s, while the dynamic transmittance was monitored at a single wavelength (the λ_max_ value determined from the spectroelectrochemistry). The chronoabsorptometry of the carbazole-based copolymers are shown in Figure 6, with the data for the fluorene-based copolymers in Figure S14. At a determined 5-s interval, P(HT-BSe-OC) possessed transmittance changes of 16.62% at 568 nm, 27.95% at 820 nm, and 43.89% at 1560 nm. For P(HoT-BSe-OC), the data were 22.06% at 590 nm, 28.47% at 910 nm, and 52.45% at 1530 nm at a 5-s interval (Figure 6). The transmittance contrasts for P(OoT-BSe-OC) were 17.18% at 590 nm and 44.27% at 1510 nm at a 5-s interval (Figure 6). At shorter switching intervals, the optical contrasts (Δ%*T*) for all of the polymers decreased significantly (Figure 7), and is exemplified by the stepped decrease in the transmittance changes of the P(HT-BSe-OC) polymer at 568 nm (Figure 7a), for which the Δ%*T* values were 18.8% at a 10-s interval, 16.62% at a 5-s interval, 9.25% at a 2-s interval, and 6.0% at a 1-s interval. The process of the doping/dedoping of the polymers is accompanied by the diffusion (in or out) process of the counter anions in the polymer matrix. The high dependence of the Δ%*T* value on the interval times indicated the sluggish diffusion rate of the counter anions in the polymer matrix.

The dynamic switching performance of the fluorene-containing polymers was also examined (Figure S14). At an interval time of 5 s, for P(HT-BSe-OF), the Δ%*T* values are 11.65% at 580 nm, 14.25% at 870 nm, and 21.29% at 1650 nm; and the data for P(HoT-BSe-OF) were 19.99% at 590 nm, 28.72% at 860 nm, and 45.89% at 1590 nm (Figure S14). The Δ%*T* values for P(OoT-BSe-OF) were found to be 12.42% at 580 nm, 23.60% at 870 nm, and 40.54% at 1510 nm (Figure S14). A step-type fall in Δ%*T* values was also found for the three fluorene-containing copolymers, which is similar to that of the three carbazole-containing polymers (Figure S15). By comparison, it was found that the Δ%*T* values at each wavelength of the fluorene-containing polymers are slightly lower than those of the carbazole-containing copolymers. From the data shown in Table 3, all of the polymers exhibited higher optical contrasts in the NIR region than in the visible light region. Therefore, these polymers might be promising candidates as active materials for the fabrication of near-infrared electrochromic devices [35].

The response time, which is defined as the time needed for 95% of the full optical switch (after which the naked eye could not sense the color change), is an important factor influencing its potential application for device fabrication [34]. In addition, the coloration efficiency (*CE*) is also an important parameter, which determines the energy utilization efficiency of the device employing the polymers as the active materials. It refers to the optical density change (Δ*OD*) per charge consumption per area (Δ*Q*) of the working electrode. The *CE* value of polymers at the specified wavelength can be calculated using the formula below [36]:

*CE* = Δ*OD*/Δ*Q*, Δ*OD* = log (*T*_b_/*T*_c_), Δ*Q* = *Q*/*A*.

where *T*_b_ and *T*_c_ are the transmittances before and after coloration, respectively; Δ*OD* is the change of the optical density, which is proportional to the amount of created color centers; and η denotes the coloration efficiency (*CE*). Δ*Q* is the amount of injected charge per unit sample area. For P(HT-BSe-OC), the response times (from the neutral to oxidized state) were 3.69 s at 568 nm, 2.9 s at 820 nm, and 3.6 s at 1560 nm, at a regular interval time of 5 s during the chronoabsorptometry measurements (Table 3). In addition, its coloration efficiencies were calculated to be 58.33 cm^2^·C^−1^ at 568 nm, 91.68 cm^2^·C^−1^ at 820 nm, and 164.65 cm^2^·C^−1^ at 1560 nm (Table 3). The *CE* values and the response times for P(HoT-BSe-OC) are 94.83 cm^2^·C^−1^ and 2.48 s at 590 nm, 144.66 cm^2^·C^−1^ and 1.14 s at 910 nm, and 149.22 cm^2^·C^−1^ and 0.6 s at 1530 nm (Table 3). For P(OoT-BSe-OC), the data were 26.30 cm^2^·C^−1^ and 1.6 s at 590 nm and 184.83 cm^2^·C^−1^ and 1.6 s at 1510 nm, respectively. As shown in Table 3, P(HoT-BSe-OC) had the best comprehensive performance among the three carbazole-based copolymers; especially, it had the highest contrast ratios at each of the respective wavelengths. The incorporation of the 3,4-dihexyloxythiophene unit in the backbone of polymer strengthened the π–π* transition of the polymer due to the strong electron-donating abilities of the alkoxy side chain, and then intensified the second π–π* transition band, which is beneficial for enhancement of the contrast ratios of the polymer in the visible region [37]. The absorption bands of the polymers in the near-infrared region is due to the generation of the polaron and bipolaron during the oxidation process, which are charged defect states, and could be stabilized by the electron-donating units (such as the thiophene derivatives). The high electron-donating ability of 3,4-dihexyloxythiophene was favorable for the generation and stabilization of these charged transitional states, which might intensify the absorption bands in the near-infrared region, and then enhance the contrast ratio in the near-infrared region. As discussed above, 3,4-dihexyloxythiophene has advantages over the other thiophene derivatives as the electron donor units in improving the optoelectronic properties of the resultant polymers. The abovementioned discussions account for the best comprehensive performance of P(HoT-BSe-OC) among the three carbazole-based polymers [38].

Table 3 also listed the switching properties of the three fluorene-containing polymers, from which it could be seen that the overall switching properties of the fluorene-containing polymers were slightly inferior to those of the carbazole-containing polymers, especially in terms of the Δ%*T* values at each of the wavelengths.

### 3.6. Colorimetry

In a simple way, the CIE 1976 L* a* b* Color Space was used to evaluate the color chromaticity of the films with a standard D65 illuminant at room atmosphere. Here, the meaning of L* represents the lightness from black to white (the range is 0–100), a* represents the relative content of red (+a*) and green (−a*), and b* represents the relative content of yellow (+b*) and blue (−b*) [37]. 

**Figure 8:**
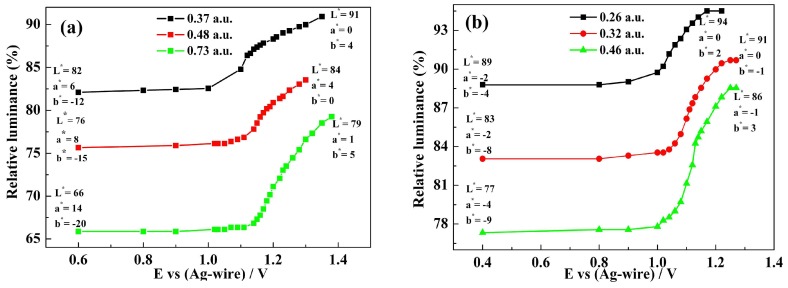

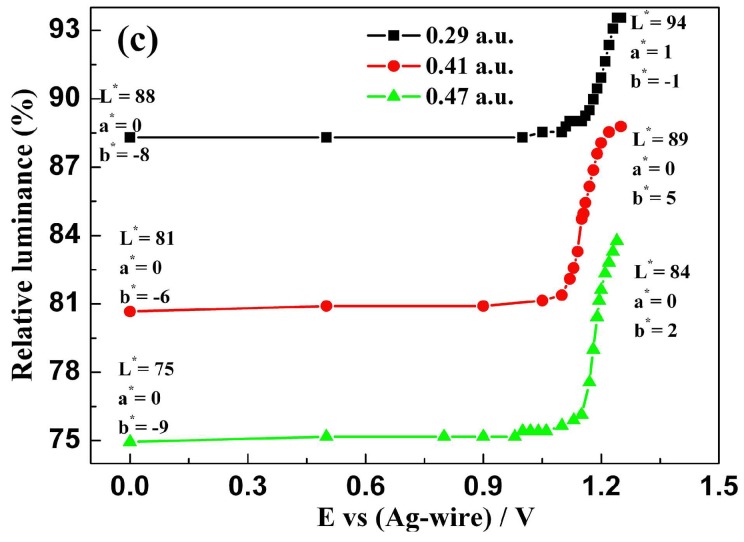
Relative luminance of polymer films as function of the externally applied potentials for three carbazole-based copolymers. (**a**) P(HT-BSe-OC); (**b**) P(HoT-BSe-OC); (**c**) P(OoT-BSe-OC).

In order to estimate the brightness of the polymer films in the process of the oxidation with the increasing applied voltage, the luminance (CIE 1976 L* values) changes were drawn in Figure 8. All of the polymers switched between the dark color of the neutral state to the translucent light color of the oxidized state. The lightness (L*) of all the polymers increased steadily during the potential switching from the neutral state to the oxidized state, indicated a bleaching process occurred during the oxidation stage.

The film thicknesses were measured by the optical densities at a determined wavelength, and the greater the absorbance, the greater the thickness. Over a series of polymer film thicknesses, the L* a* b* color values of all of the polymers experienced a similar tendency (Figure 8). For P(HT-BSe-OC), the luminance values varied from 82 to 91 (absorption maximum = 0.37 a.u.), from 76 to 84 (absorption maximum = 0.48 a.u.), and from 66 to 79 (absorption maximum= 0.73 a.u.) (Figure 8a). The a* values of polymer P(HT-BSe-OC) changed from 6 to 0 (0.37 absorbance), from 8 to 4 (0.48 absorbance), and from 14 to 1 (0.73 absorbance), showing the reduction in the red element of the color, which is consistent with the disappearance of the absorption band centered at 568 nm. While the b* values of P(HT-BSe-OC) changed in a larger range, from −12 to 4 (0.37 a.u absorbance), from −15 to 0 (0.48 a.u absorbance), and from −20 to 5 (0.73 a.u absorbance), indicating the reduction in the blue element and the increase in the yellow element (Figure 8a). As for P(HoT-BSe-OC) films with different thickness, the variation trend of the L* a* b* values was consistent with that of P(HT-BSe-OC); specifically speaking, the brightness, red, and yellow elements increased, and green and blue elements are weakened (Figure 8b). The L* a* b* values of P(OoT-BSe-OC) experienced a similar course to the above two carbazole-containing copolymers, which is presented in Figure 8c. The relative luminance changes of three fluorene-containing copolymers are depicted in Figure S16 (see Supporting Information), and the basic rule was also that the values of L* and b* were increased and the a* values were reduced.

## 4. Conclusions

In general, six new D-A-type polymers containing benzoselenadiazole (BSe) as the acceptor unit were synthesized via the simple Suzuki-Miyaura reaction. The variables in the polymer backbone are five electron-donating units, namely 3-hexylthiophene, 3,4-dihexyloxythiophene, 3,4-dioctyloxythiophene, 9-(heptadecan-9-yl)-9*H*-carbazole, and 9-(heptadecan-9-yl)-9*H*-fluorene. The introduction of side alkyl or alkoxy chains to the polymer backbone makes the polymers soluble in chloroform and processable for spray-film formation. Compared with 3-hexylthiophene, the introduction of the 3,4-dialkoxythiophene to the polymer backbone, which has much stronger electron-donating abilities than the former, would lead to the improvement in the performance of the polymers, including the reduction in initial oxidation potentials and the bandgaps as well as the improvement of the electrochromic properties of the polymers. Meanwhile, the steric repulsion hindrance effects of the neighboring side chains will drive the performance of the polymers in the opposite direction; for example, the rise in bandgap and the indemnification of electrochromic properties. At last, it was found that using either carbazole or fluorene as the electron-donating units along the polymer backbone had nearly no obvious effects on the comprehensive properties of the resultant polymers.

## Figures and Tables

**Table 1:** Synthetic yield, molecular weight, polydispersity, and thermal data of polymers.

Polymer	Yield (%)	^b^ *M*_n_(kDa)	^a^ *M*_w_ (kDa)	PDI	^c^ *T*_d_ (°C)
P(HT-BSe-OC)	84	7.6	16.9	2.2	359
P(HT-BSe-OF)	81	7.8	16.2	2.1	398
P(HoT-BSe-OC)	78	7.0	14.3	2.0	340
P(HoT-BSe-OF)	89	6.7	15.6	2.3	335
P(OoT-BSe-OC)	86	6.7	14.3	2.1	334
P(OoT-BSe-OF)	82	6.5	15.4	2.4	265

^a^ *M*_w_: The weight-average molecular weight; ^b^ *M*_n_: the number-average molecular weight; ^c^ *T*_d_: the decomposition temperature at which 5% weight loss occurs; PDI: polydispersity index.

**Table 2:** Electrochemical and optical properties of polymers.

Polymer	*E*_onset_, vs. (Ag-wire)/V	Absorption, λ_max_/nm		Neutral state			
		Solution	Film	λ_onset_/ nm	^a^ *E*_g,opt_/ eV	^b^ HOMO/eV	^c^ LUMO/eV
P(HT-BSe-OC)	1.15	371,547	377,568	698	1.78	−5.57	−3.79
P(HoT-BSe-OC)	1.03	396,572	402,592	724	1.71	−5.45	−3.74
P(OoT-BSe-OC)	1.04	390,561	399,582	713	1.74	−5.46	−3.72
P(HT-BSe-OF)	1.14	372,545	368,582	709	1.75	−5.56	−3.81
P(HoT-BSe-OF)	0.995	397,569	403,590	721	1.72	−5.42	−3.69
P(OoT-BSe-OF)	1.13	392,562	398,579	713	1.74	−5.55	−3.81

^a^ The optical band gaps (*E*_g,op_) were calculated from *E*_g,opt_ = 1241/λ_onset_; ^b^ HOMO = −e(*E*_onset_ + 0.02 + 4.4) (the number 0.02 is a correction parameter versus the ferrocene redox couple) [16]; ^c^ LUMO = HOMO + *E*_g,opt_ [25]. HOMO: highest occupied molecular orbital; LUMO: lowest unoccupied molecular orbital.

**Table 3:** The dynamic switching properties of the six polymers.

^a^ Polymers	λ (nm)	Optical contrast (Δ*T*%)	Response time (s)	Coloration efficiency (CE, cm^2^·C^−1^)
P(HT-BSe-OC)	568	16.22	3.69	58.53
	820	27.95	2.9	91.68
	1560	43.89	3.6	164.65
P(HoT-BSe-OC)	590	22.06	2.48	94.83
	910	28.47	1.14	144.66
	1530	52.45	0.6	149.22
P(OoT-BSe-OC)	590	17.18	2.9	26.30
	1510	44.27	1.6	184.83
P(HT-BSe-OF)	580	11.65	2.08	68.33
	870	14.25	1.9	53.26
	1650	21.29	0.81	194.79
P(HoT-BSe-OF)	590	19.99	1.73	116.45
	860	28.72	0.26	141.84
	1590	45.89	0.15	218.55
P(OoT-BSe-OF)	580	12.42	2.65	99.31
	870	23.60	1.48	135.10
	1510	40.54	1.21	182.74

^a^ All polymers tested have a similar thickness of absorbance (a.u.) = 0.46, and the retention time used in the chronoabsorptometry was 5 s.

## Supplementary Materials

The following are available online at http://www.mdpi.com/2073-4360/10/4/450/s1. Figure S1. The ^1^H NMR (upper) and ^13^C NMR (under) of 4,7-bis(4-hexylthiophen-2-yl)benzo[c][1,2,5]selenadiazole (HT-BSe). *x* refers to the peak of CDH_3_ (solvent), *y* refers to the peak of H_2_O, *z* refers to the peak of tetramethylsilane (internal standard substance). Figure S2. The ^1^H NMR (upper) and ^13^C NMR (under) of 4,7-bis(3,4-bis(hexyloxy)thiophen-2-yl)benzo[c][1,2,5]selenadiazole (HoT-BSe). *x* refers to the peak of CDH_3_ (solvent), *y* refers to the peak of H_2_O, *z* refers to the peak of tetramethylsilane (internal standard substance). Figure S3. The ^1^H NMR (upper) and ^13^C NMR (under) of 4,7-bis(3,4-bis(octyloxy)thiophen-2-yl)benzo[c][1,2,5]selenadiazole (HoT-BSe). *x* refers to the peak of CDH_3_ (solvent), *y* refers to the peak of H_2_O, *z* refers to the peak of tetramethylsilane (internal standard substance). Figure S4. The ^1^H NMR (upper) and ^13^C NMR (under) of 4,7-bis(5-bromo-4-hexylthiophen-2-yl)benzo[c][1,2,5]selenadiazole (2Br-HT-BSe). *x* refers to the peak of CDH_3_ (solvent), *y* refers to the peak of H_2_O, *z* refers to the peak of tetramethylsilane (internal standard substance). Figure S5: The ^1^H NMR (upper) and ^13^C NMR (under) of 4,7-bis(5-bromo-3,4-bis(hexyloxy)thiophen-2-yl)benzo[c][1,2,5]selenadia-zole (2Br-HoT-BSe). *x* refers to the peak of CDH_3_ (solvent), *y* refers to the peak of H_2_O, *z* refers to the peak of tetramethylsilane (internal standard substance). Figure S6. The ^1^H NMR (upper) and ^13^C NMR (under) of 4,7-bis(5-bromo-3,4-bis(octyloxy)thiophen-2-yl)benzo[c][1,2,5]selenadia-zole (2Br-OoT-BSe). *x* refers to the peak of CDH_3_ (solvent), *y* refers to the peak of H_2_O, *z* refers to the peak of tetramethylsilane (internal standard substance). Figure S7. The ^1^H NMR of P(HT-BSe-OC). *x* refers to the peak of CDH_3_ (solvent), *y* refers to the peak of H_2_O, *z* refers to the peak of tetramethylsilane (internal standard substance). Figure S8. The ^1^H NMR of P(HoT-BSe-OC). *x* refers to the peak of CDH_3_ (solvent), *y* refers to the peak of H_2_O, *z* refers to the peak of tetramethylsilane (internal standard substance). Figure S9. The ^1^H NMR of P(OoT-BSe-OC). *x* refers to the peak of CDH_3_ (solvent), *y* refers to the peak of H_2_O, *z* refers to the peak of tetramethylsilane (internal standard substance). Figure S10. The ^1^H NMR of P(HT-BSe-OF). *x* refers to the peak of CDH_3_ (solvent), *y* refers to the peak of H_2_O, *z* refers to the peak of tetramethylsilane (internal standard substance). Figure S11. The ^1^H NMR of P(HoT-BSe-OF). *x* refers to the peak of CDH_3_ (solvent), *y* refers to the peak of H_2_O, *z* refers to the peak of tetramethylsilane (internal standard substance). Figure S12. The ^1^H NMR of P(OoT-BSe-OF). *x* refers to the peak of CDH_3_ (solvent), *y* refers to the peak of H_2_O, *z* refers to the peak of tetramethylsilane (internal standard substance). Figure S13. Spectroelectrochemical spectra of thee fluorene-based copolymers. (**a**) P(HT-BSe-OF); (**b**) P(HoT-BSe-OF); (**c**) P(OoT-BSe-OF). Figure S14. The chronoabsorptometry of three fluorene-based polymers with the interval times of 5 s in the square wave potential method. The test wavelengths and the corresponding contrast ratios are labeled in the figures. (**a**) P(HT-BSe-OF); (**b**) P(HoT-BSe-OF); (**c**) P(OoT-BSe-OF). Figure S15. The dependence of the optical contrast ratios on the interval times in the chronoabsorptometry study. The interval times tested in the in the square wave potential method varied at 10, 5, 2, and 1 s in turn. The test wavelengths and the corresponding contrast ratios are labeled in the figures. (**a**–**c**), P(HT-BSe-OF); (**d**–**f**): P(HoT-BSe-OF); (**g**–**i**): P(OoT-BSe-OF). Figure S16. Relative luminance of polymer films as function of the externally applied potentials for three carbazole-based copolymers. (**a**) P(HT-BSe-OF); (**b**) P(HoT-BSe-OF); (**c**) P(OoT-BSe-OF).
