# Supplementary material for: Soluble Electrochromic Polymers Incorporating Benzoselenadiazole and Electron Donor Units (Carbazole or Fluorene): Synthesis and Electronic-Optical Properties
Source: Polymers (Basel). 2018 Apr 17;10(4):450. doi: 10.3390/polym10040450 (PMC6415458; doi:10.3390/polym10040450)
Supplement: Supplementary file 1 [file polymers-10-00450-s001.pdf]

polymers

MDPI

Electronic Supplementary Materials

Soluble electrochromic polymers incorporating benzoselenadiazole and electron acceptor units (carbazole or fluorene): synthesis and electronic-optical properties

Jianzhong Xu [1], Qi Ji [1,2], Lingqian Kong [3], Hongmei Du [2], Xiuping Ju [3] and Jinsheng Zhao [2,*]

[1] College of Chemistry and Environmental Science, Hebei University, Baoding 071002, China; xujianzhong@126.com; (J.X.); wangyan@lcu.edu.cn (Q.J.)
[2] Department of Chemistry and Chemical Engineering, Liaocheng University, Liaocheng 252059, China; duhongmei@lcu.edu.cn (H. Du)
[3] Dongchang Colledge, Liaocheng University, Liaocheng 252059, China; lingqiankong@126.com (L.K.); jxp1127@163.com (X.J.)
* Correspondence: zhaojinsheng@lcu.edu.cn; Tel.: +86-635-853-9607

Received: 25 March 2018; Accepted: 14 April 2018; Published: date

the Content of The Electronic Supplementary Materials:

1. FigureS1. The ^{1}H NMR (upper) and ^{13}C NMR (under) of 4,7-bis(4-hexylthiophen-2-yl)benzo[c][1,2,5]selenadiazole (HT-BSe). x refers to the peak of CDH_3 (solvent), y refers to the peak of H_2O, z refers to the peak of tetramethylsilane (internal standard substance).
2. FigureS2. The ^{1}H NMR (upper) and ^{13}C NMR (under) of 4,7-bis(3,4-bis(hexyloxy)thiophen-2-yl)benzo[c][1,2,5]selenadiazole (HoT-BSe). x refers to the peak of CDH_3 (solvent), y refers to the peak of H_2O, z refers to the peak of tetramethylsilane (internal standard substance).
3. FigureS3. The ^{1}H NMR (upper) and ^{13}C NMR (under) of 4,7-bis(3,4-bis(octyloxy)thiophen-2-yl)benzo[c][1,2,5]selenadiazole (HoT-BSe). x refers to the peak of CDH_3 (solvent), y refers to the peak of H_2O, z refers to the peak of tetramethylsilane (internal standard substance).
4. FigureS4. The ^{1}H NMR (upper) and ^{13}C NMR (under) of 4,7-bis(5-bromo-4-hexylthiophen-2-yl)benzo[c][1,2,5]selenadiazole (2Br-HT-BSe). x refers to the peak of CDH_3 (solvent), y refers to the peak of H_2O, z refers to the peak of tetramethylsilane (internal standard substance).
5. FigureS5 The ^{1}H NMR (upper) and ^{13}C NMR (under) of 4,7-bis(5-bromo-3,4-bis(hexyloxy)thiophen-2-yl)benzo[c][1,2,5]selenadia-zole (2Br-HoT-BSe). x refers to the peak of CDH_3 (solvent), y refers to the peak of H_2O, z refers to the peak of tetramethylsilane (internal standard substance).
6. FigureS6. The ^{1}H NMR (upper) and ^{13}C NMR (under) of 4,7-bis(5-bromo-3,4-bis(octyloxy)thiophen-2-yl)benzo[c][1,2,5]selenadia-zole (2Br-OoT-BSe). x refers to the peak of CDH_3 (solvent), y refers to the peak of H_2O, z refers to the peak of tetramethylsilane (internal standard substance).
7. FigureS7. The ^{1}H NMR of P(HT-BSe-OC). x refers to the peak of CDH_3 (solvent), y refers to the peak of H_2O, z refers to the peak of tetramethylsilane (internal standard substance).
8. Figure S8. The ^{1}H NMR of P(HoT-BSe-OC). x refers to the peak of CDH_3 (solvent), y refers to the peak of H_2O, z refers to the peak of tetramethylsilane (internal standard substance).
9. FigureS9. The ^{1}H NMR of P(OoT-BSe-OC). x refers to the peak of CDH_3 (solvent), y refers to the peak of H_2O, z refers to the peak of tetramethylsilane (internal standard substance).
10. FigureS10. The ^{1}H NMR of P(HT-BSe-OF). x refers to the peak of CDH_3 (solvent), y refers to the peak of H_2O, z refers to the peak of tetramethylsilane (internal standard substance).
11. FigureS11. The ^{1}H NMR of P(HoT-BSe-OF). x refers to the peak of CDH_3 (solvent), y refers to the peak of H_2O, z refers to the peak of tetramethylsilane (internal standard substance).

12. FigureS12. The ^{1}H NMR of P(OoT-BSe-OF). x refers to the peak of CDH_3 (solvent), y refers to the peak of H_2O, z refers to the peak of tetramethylsilane (internal standard substance).

13. FigureS13. Spectroelectrochemical spectra of thee fluorene based copolymers. (a), P(HT-BSe-OF), (b) P(HoT-BSe-OF), (c) P(OoT-BSe-OF).

14. FigureS14. The chronoabsorptometry of three fluorene based polymers with the interval times of 5 s in the square wave potential method. The test wavelengths and the corresponding contrast ratios are labeled in the figures. (a), P(HT-BSe-OF); (b) P(HoT-BSe-OF); (c) P(OoT-BSe-OF).

15. FigureS15. The dependence of the optical contrast ratios on the interval times in the chronoabsorptometry study. The interval times stetted in the in the square wave potential method varied at 10 s, 5 s, 2 s, 1 s in turn. The test wavelengths and the corresponding contrast ratios are labeled in the figures. (a-c), P(HT-BSe-OF); (d-f): P(HoT-BSe-OF); (g-i): P(OoT-BSe-OF).

16. FigureS16. Relative luminance of polymer films as function of the externally applied potentials for three carbazole based copolymers. (a) P(HT-BSe-OF); (b) P(HoT-BSe-OF); (c) P(OoT-BSe-OF).

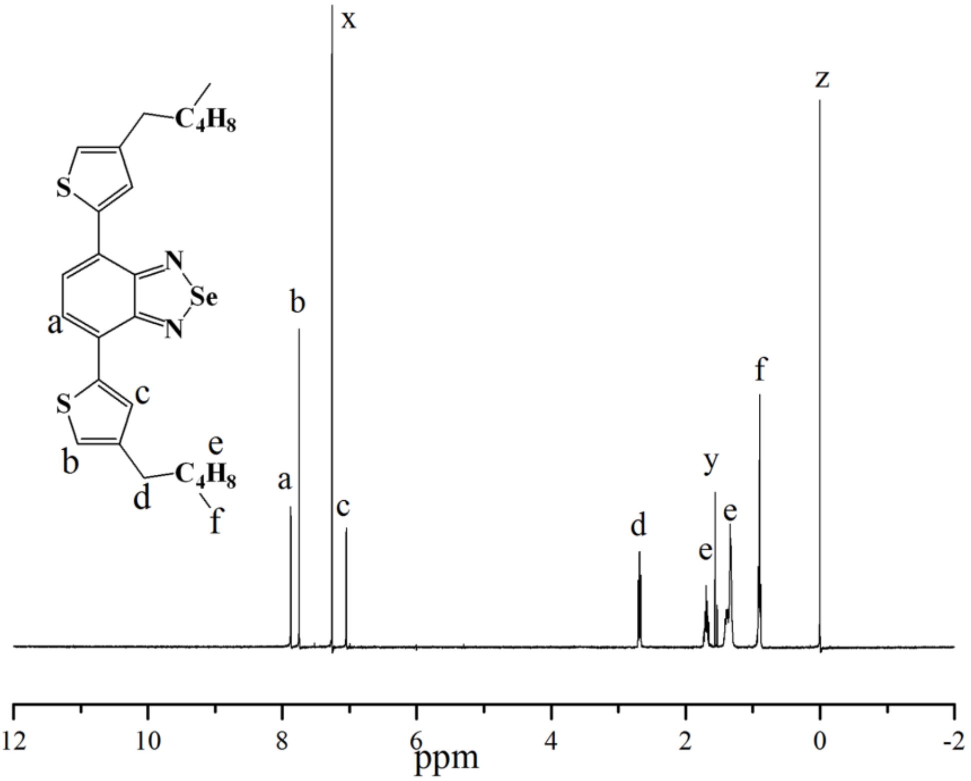

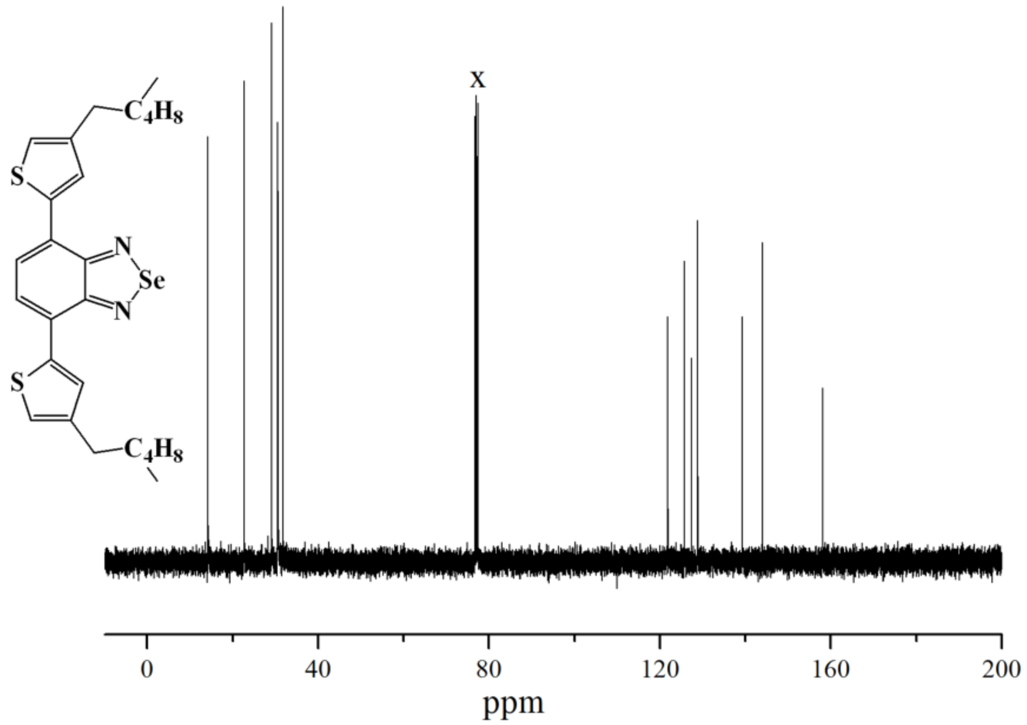

Figure S1. The ^{1}H NMR (upper) and ^{13}C NMR (under) of 4,7-bis(4-hexylthiophen-2-yl)benzo[c][1,2,5]selenadiazole (HT-BSe). x refers to the peak of CDH_3 (solvent), y refers to the peak of H_2O, z refers to the peak of tetramethylsilane (internal standard substance).

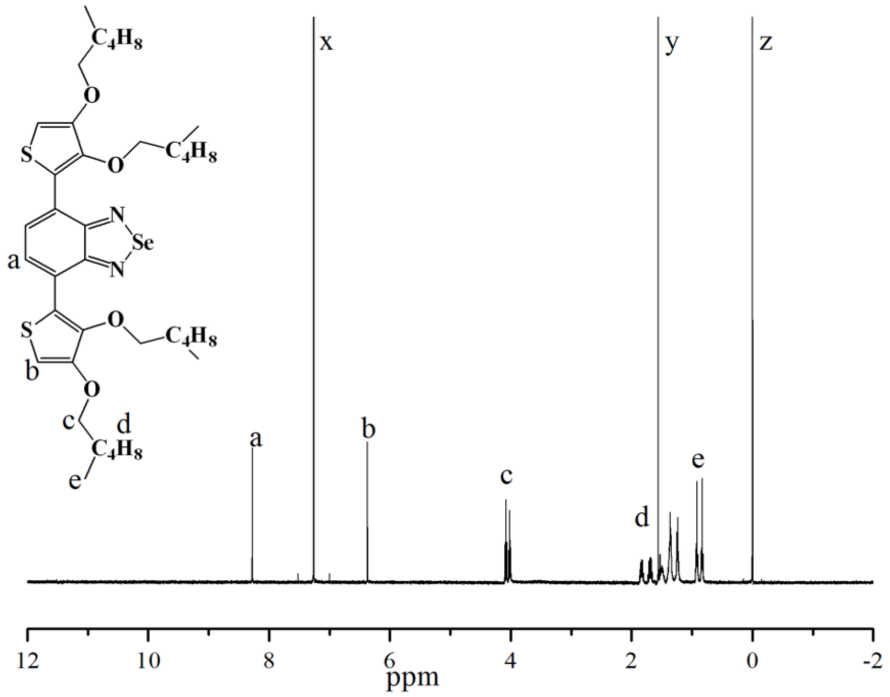

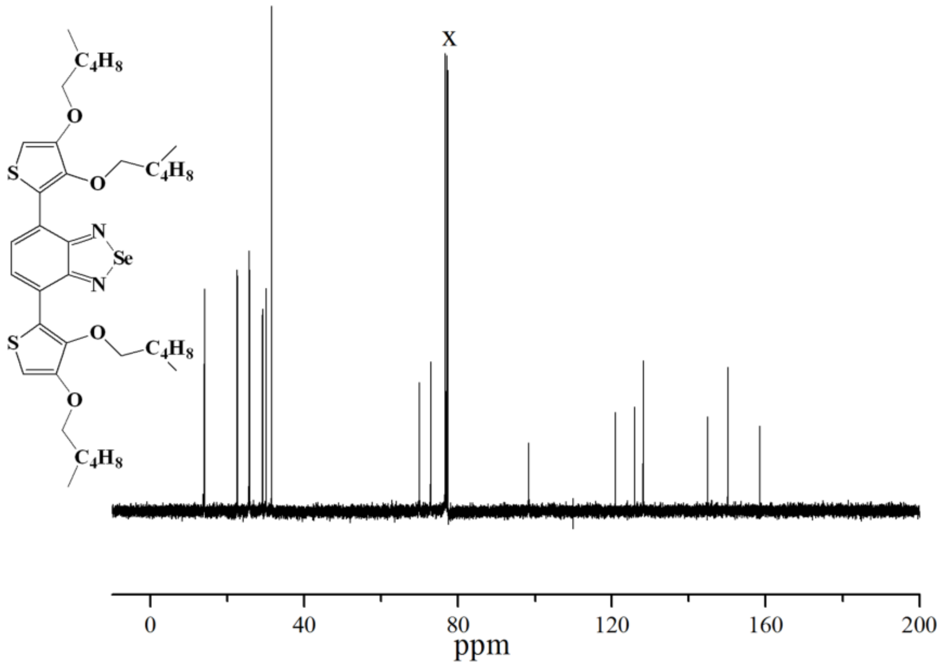

Figure S2. The ^{1}H NMR (upper) and ^{13}C NMR (under) of 4,7-bis(3,4-bis(hexyloxy)thiophen-2-yl)benzo[c][1,2,5]selenadiazole (HoT-BSe). x refers to the peak of CDH_3 (solvent), y refers to the peak of H_2O, z refers to the peak of tetramethylsilane (internal standard substance).

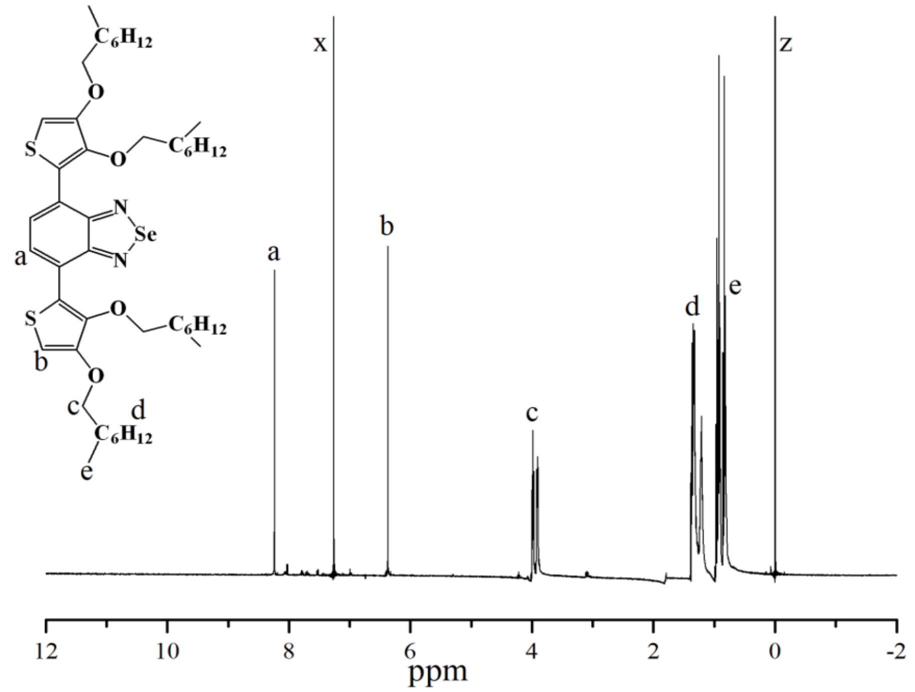

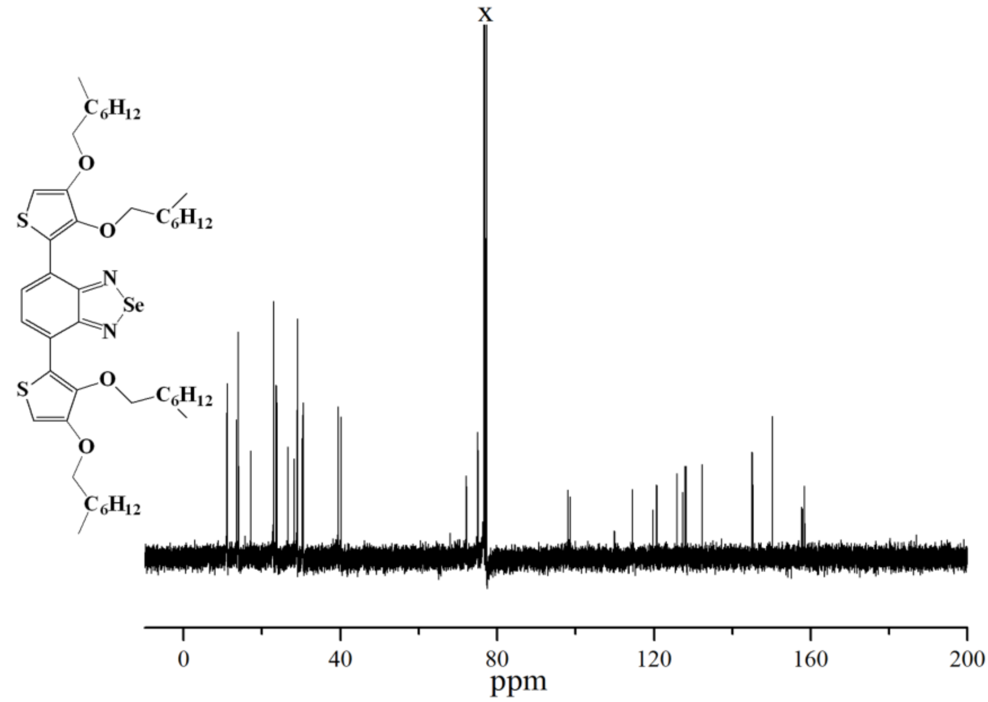

Figure S3. The ^{1}H NMR (upper) and ^{13}C NMR (under) of 4,7-bis(3,4-bis(octyloxy)thiophen-2-yl)benzo[c][1,2,5]selenadiazole (HoT-BSe). x refers to the peak of CDH_3 (solvent), y refers to the peak of H_2O, z refers to the peak of tetramethylsilane (internal standard substance).

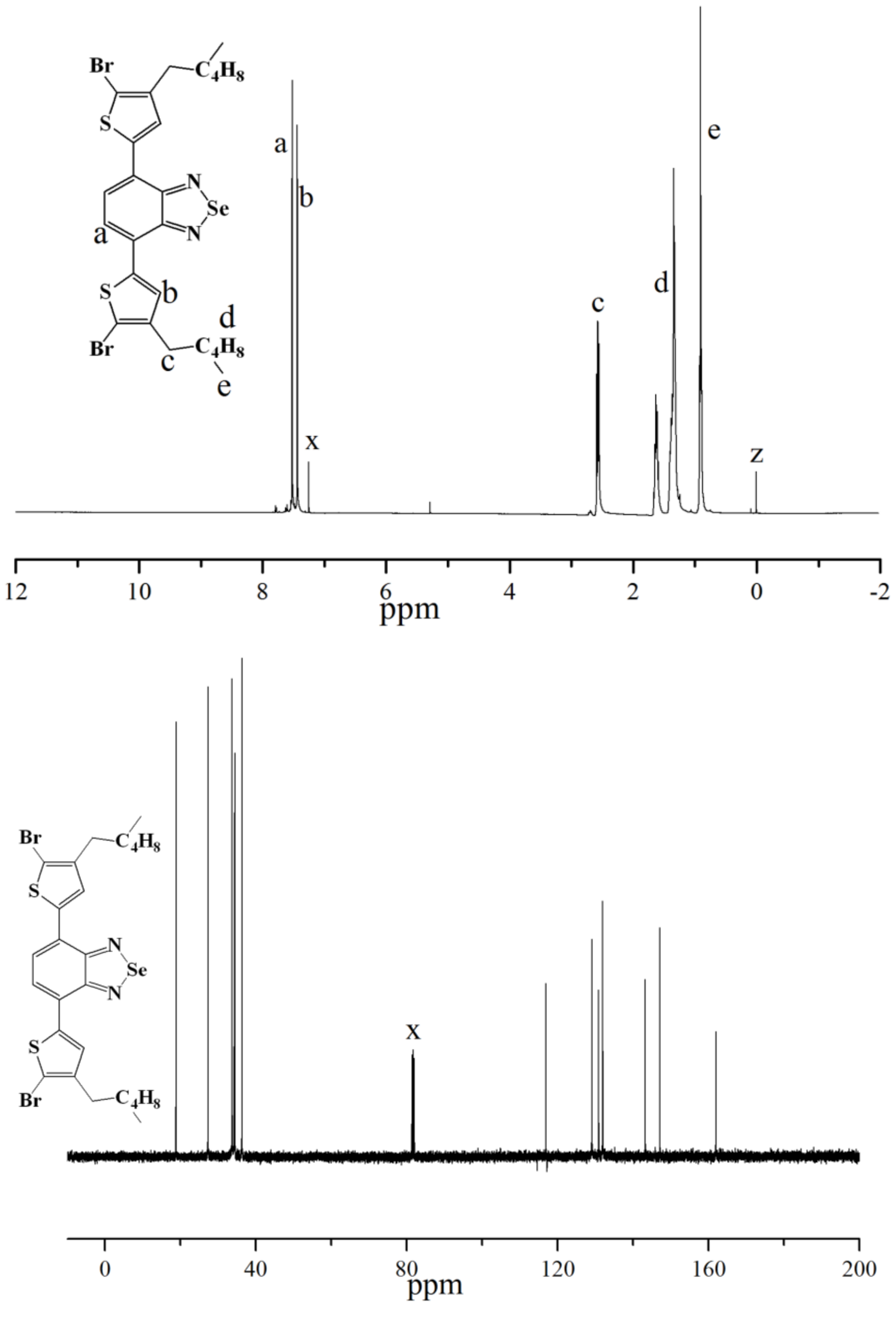

Figure S4. The ^{1}H NMR (upper) and ^{13}C NMR (under) of 4,7-bis(5-bromo-4-hexylthiophen-2-yl)benzo[c][1,2,5]selenadiazole (2Br-HT-BSe). x refers to the peak of CDH_3 (solvent), y refers to the peak of H_2O, z refers to the peak of tetramethylsilane (internal standard substance).

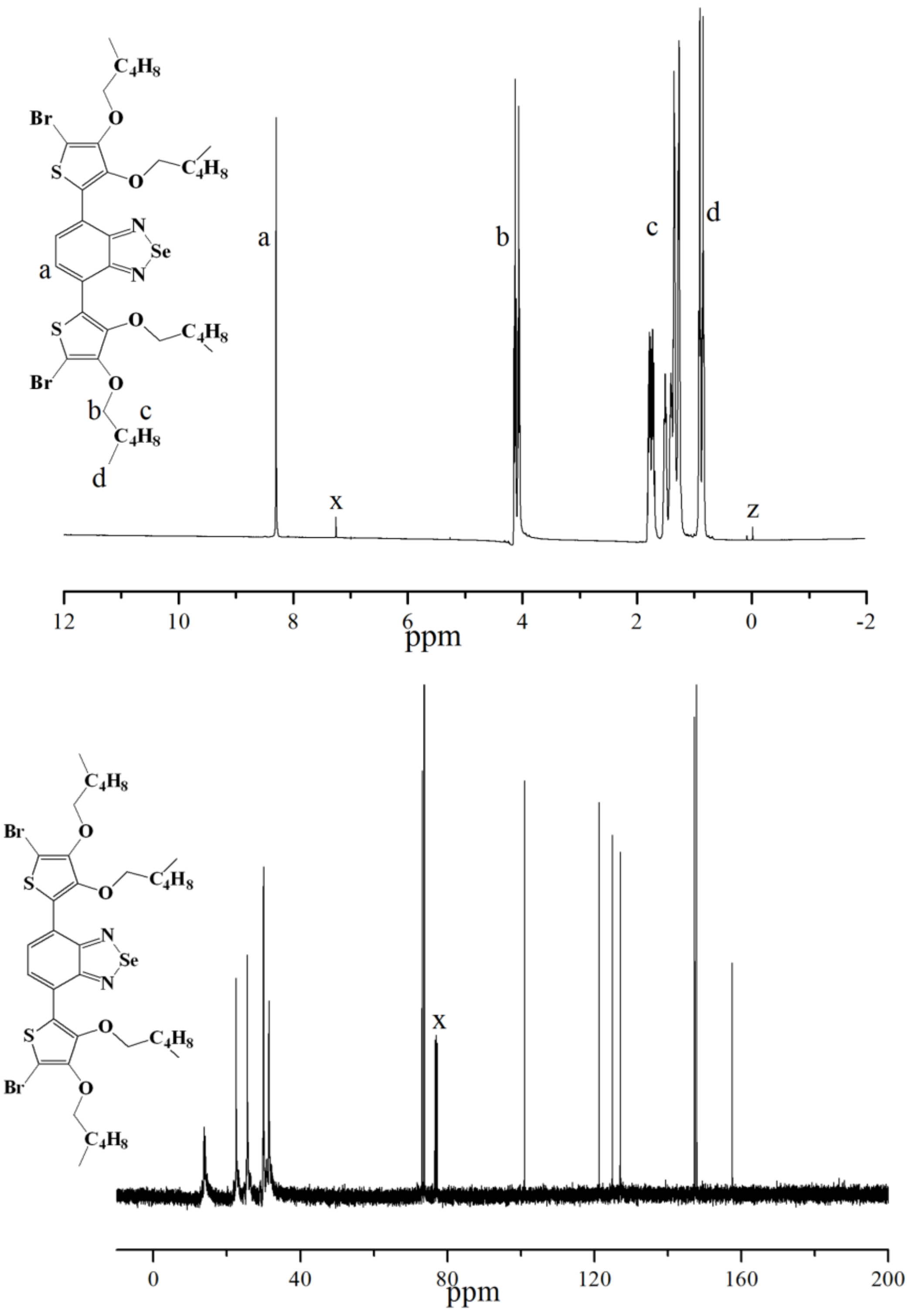

Figure S5 The ^{1}H NMR (upper) and ^{13}C NMR (under) of 4,7-bis(5-bromo-3,4-bis(hexyloxy)thiophen-2-yl)benzo[c][1,2,5]selenadia-zole (2Br-HoT-BSe). x refers to the peak of CDH_3 (solvent), y refers to the peak of H_2O, z refers to the peak of tetramethylsilane (internal standard substance).

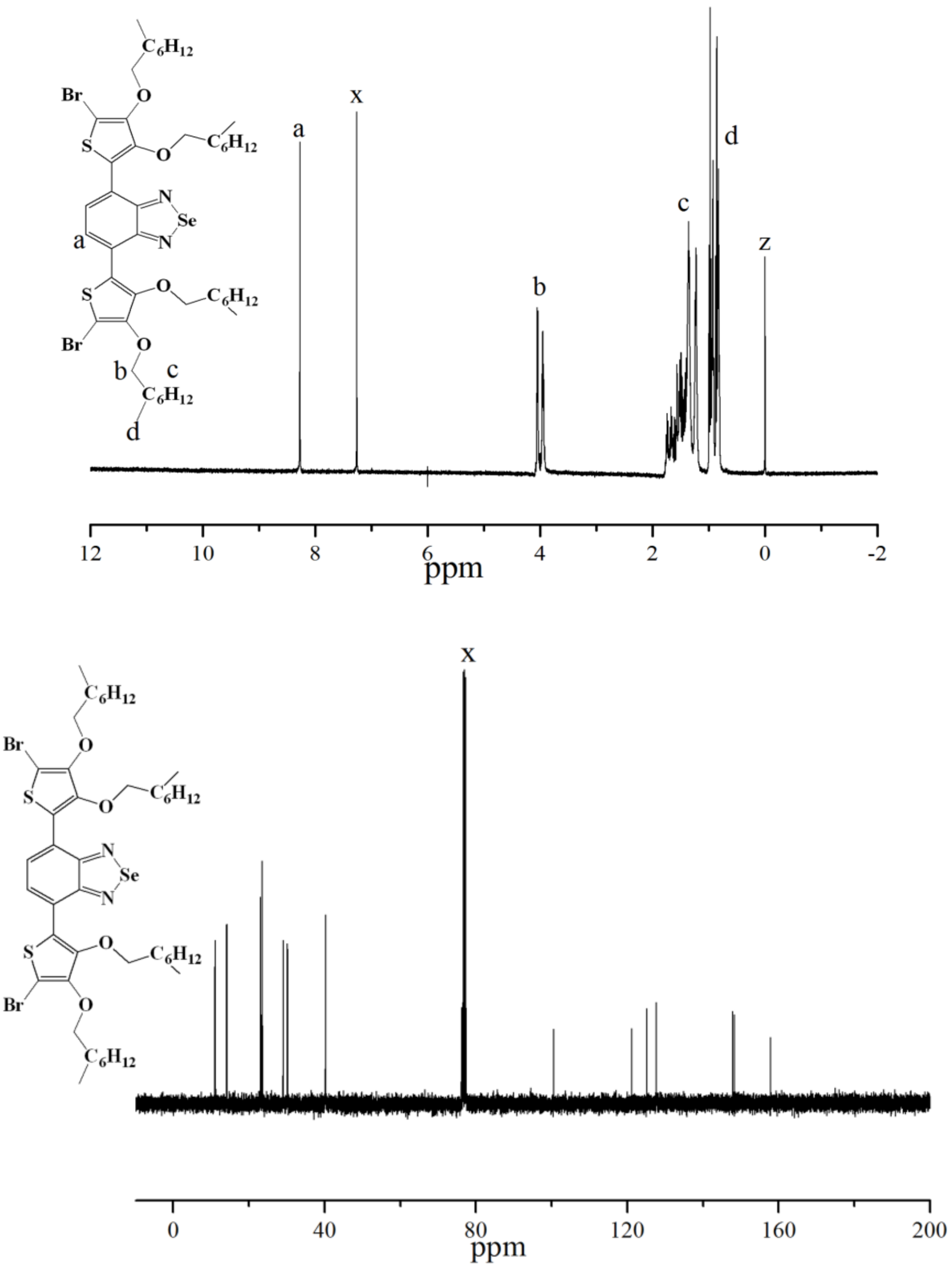

Figure S6. The ^{1}H NMR (upper) and ^{13}C NMR (under) of 4,7-bis(5-bromo-3,4-bis(octyloxy)thiophen-2-yl)benzo[c][1,2,5]selenadia-zole (2Br-OoT-BSe). x refers to the peak of CDH_3 (solvent), y refers to the peak of H_2O, z refers to the peak of tetramethylsilane (internal standard substance).

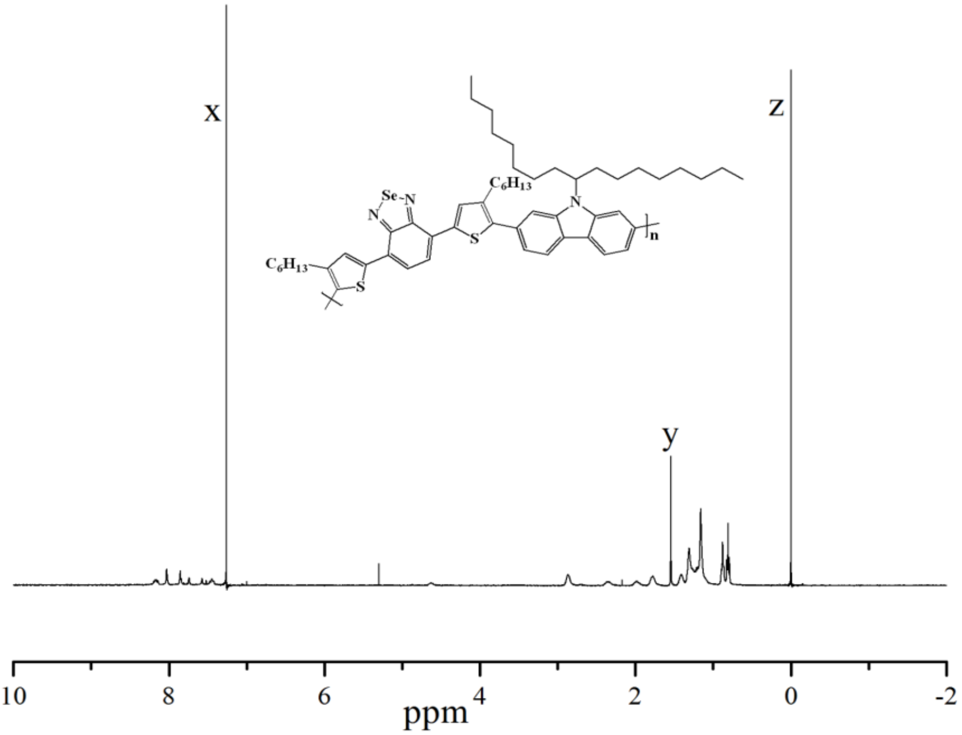

Figure S7. The ^{1}H NMR of P(HT-BSe-OC). x refers to the peak of CDH_3 (solvent), y refers to the peak of H_2O, z refers to the peak of tetramethylsilane (internal standard substance).

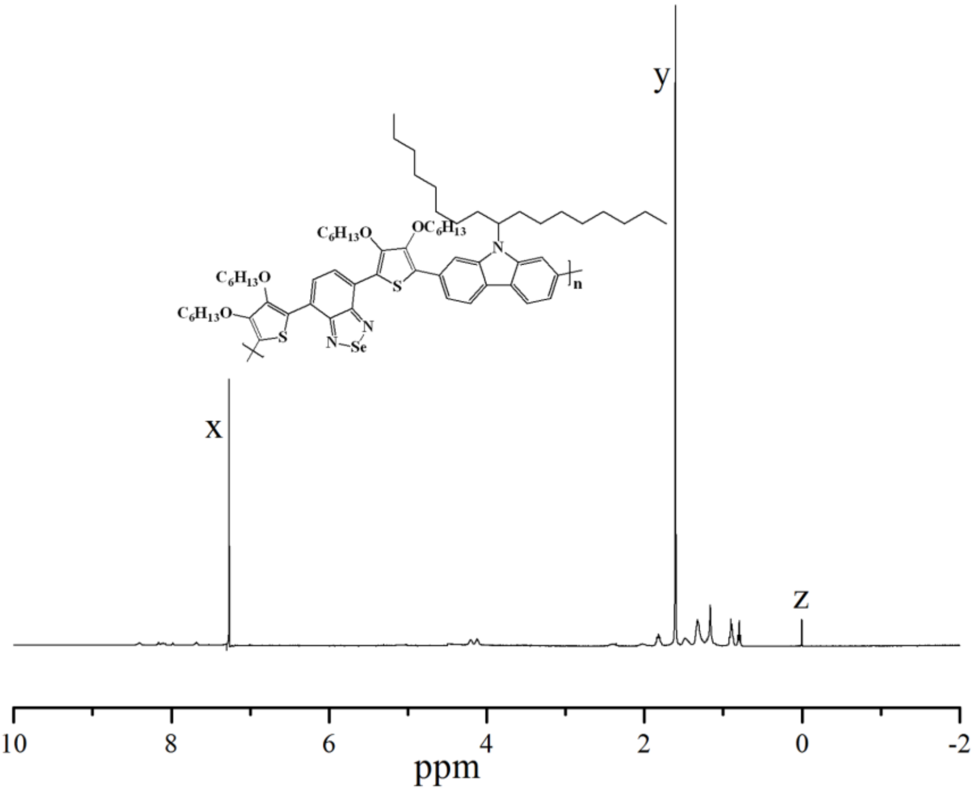

Figure S8. The ^{1}H NMR of P(HoT-BSe-OC). x refers to the peak of CDH_3 (solvent), y refers to the peak of H_2O, z refers to the peak of tetramethylsilane (internal standard substance).

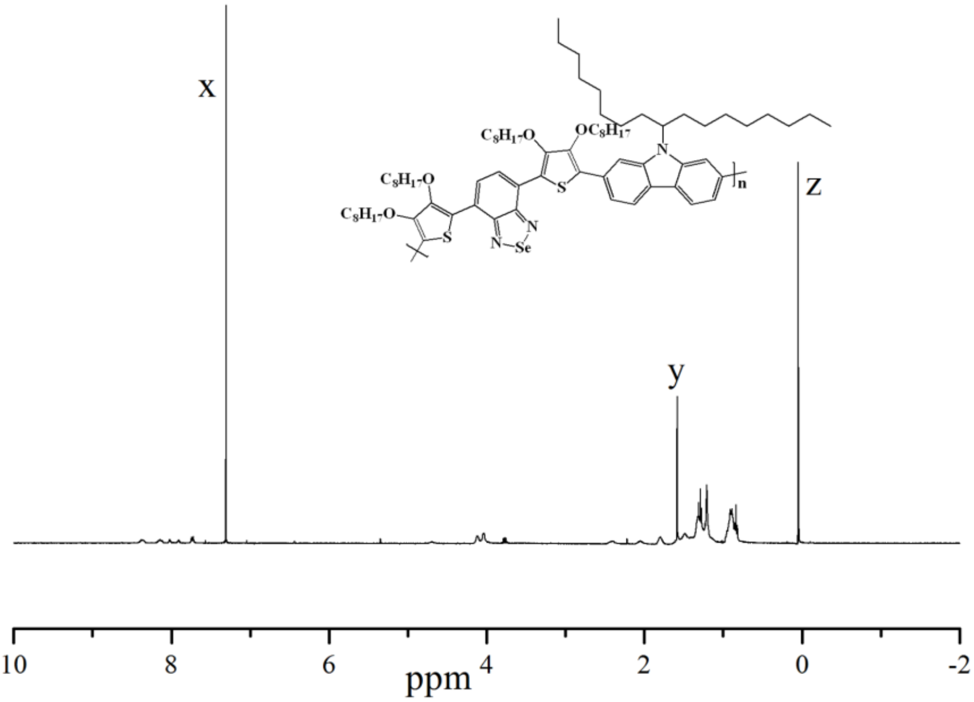

Figure S9. The ^{1}H NMR of P(OoT-BSe-OC). x refers to the peak of CDH_3 (solvent), y refers to the peak of H_2O, z refers to the peak of tetramethylsilane (internal standard substance).

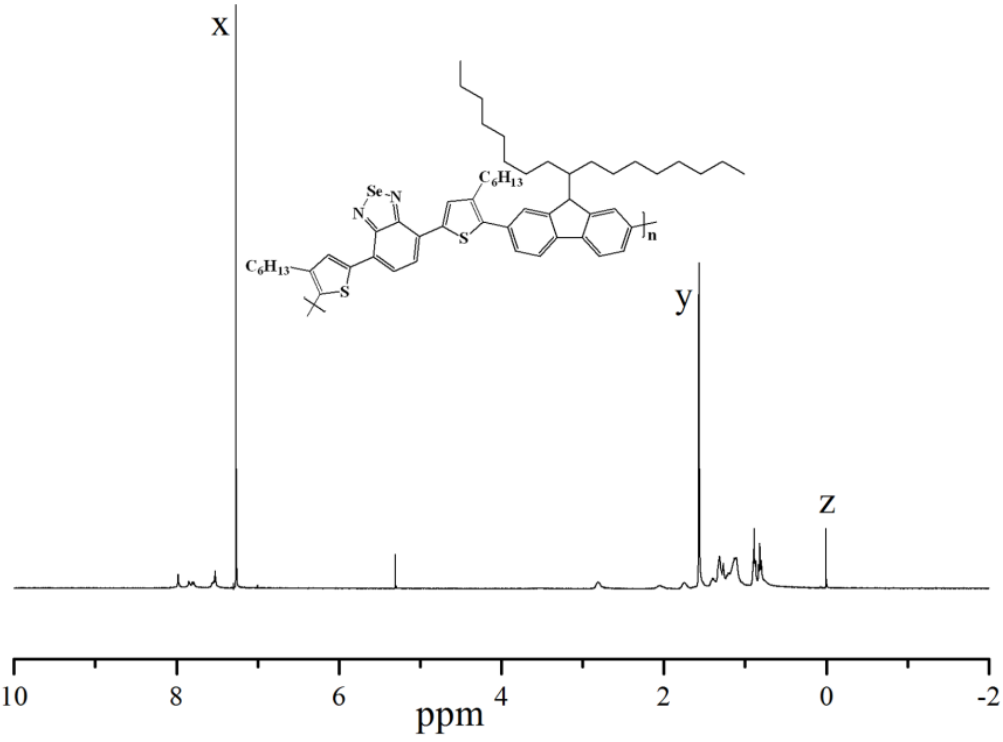

Figure S10. The ^{1}H NMR of P(HT-BSe-OF). x refers to the peak of CDH_3 (solvent), y refers to the peak of H_2O, z refers to the peak of tetramethylsilane (internal standard substance).

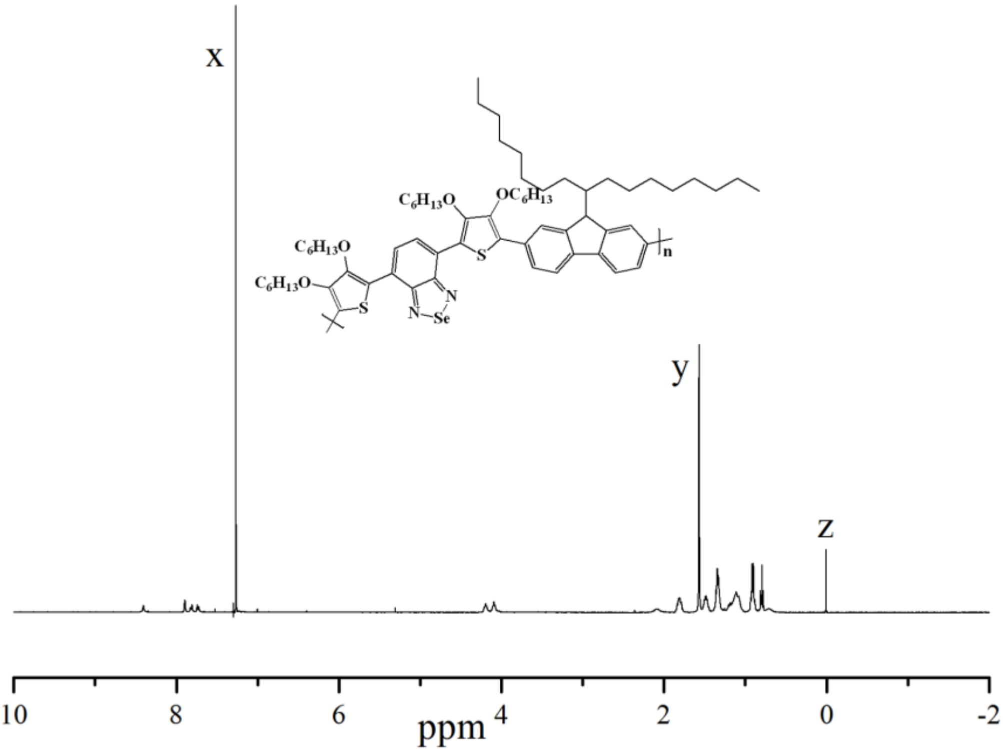

Figure S11. The ^{1}H NMR of P(HoT-BSe-OF). x refers to the peak of CDH_3 (solvent), y refers to the peak of H_2O, z refers to the peak of tetramethylsilane (internal standard substance).

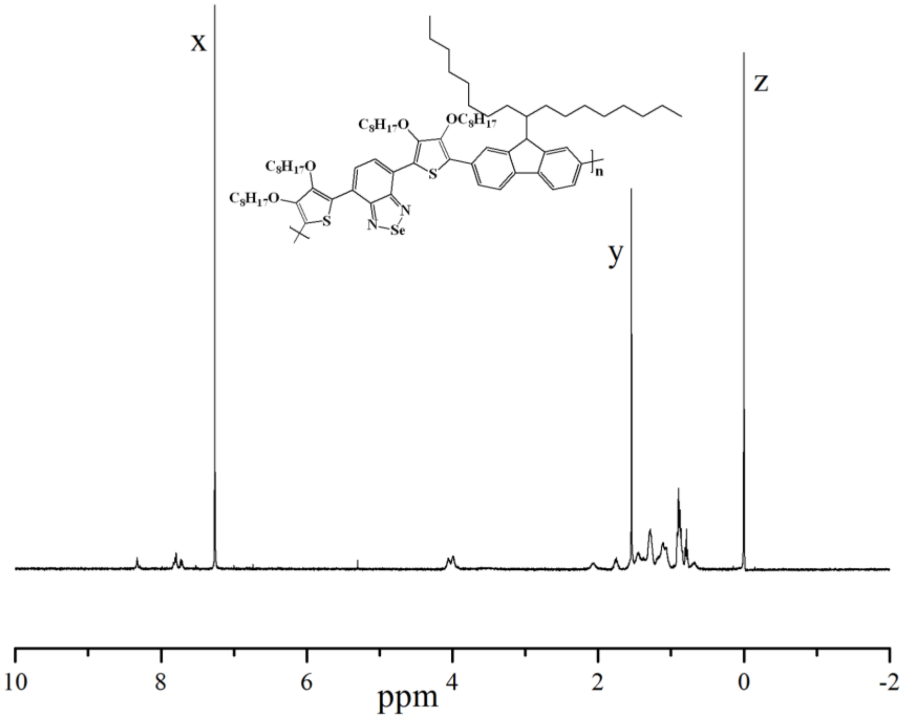

Figure S12. The ^{1}H NMR of P(OoT-BSe-OF). x refers to the peak of CDH_3 (solvent), y refers to the peak of H_2O, z refers to the peak of tetramethylsilane (internal standard substance).

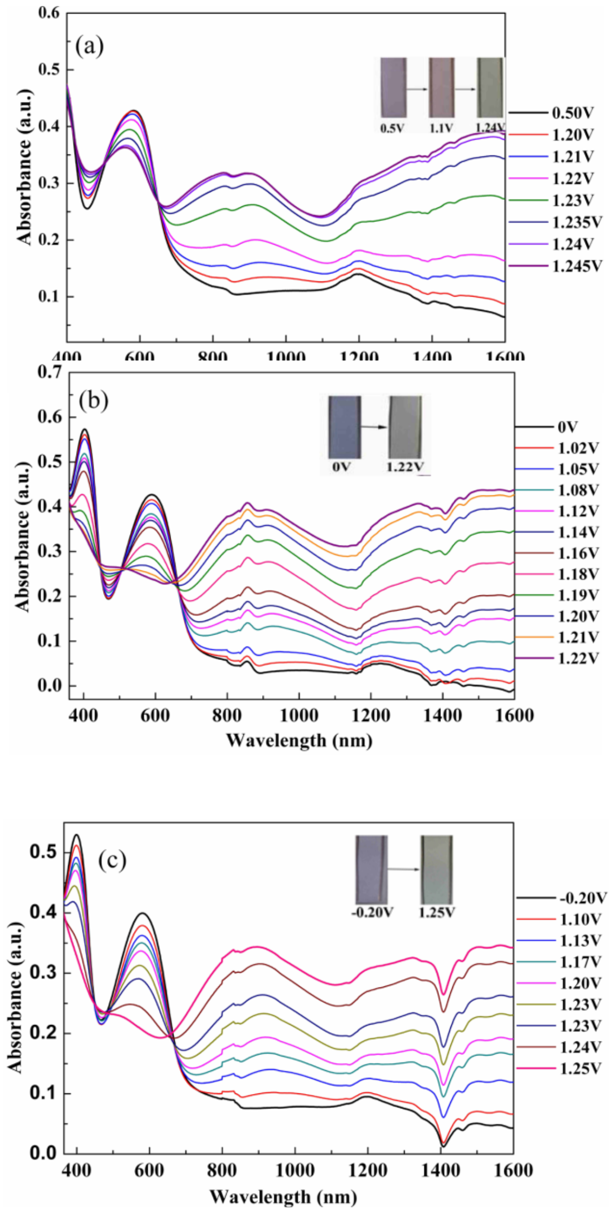

Figure S13. Spectroelectrochemical spectra of thee fluorene based copolymers. (a), P(HT-BSe-OF), (b) P(HoT-BSe-OF), (c) P(OoT-BSe-OF).

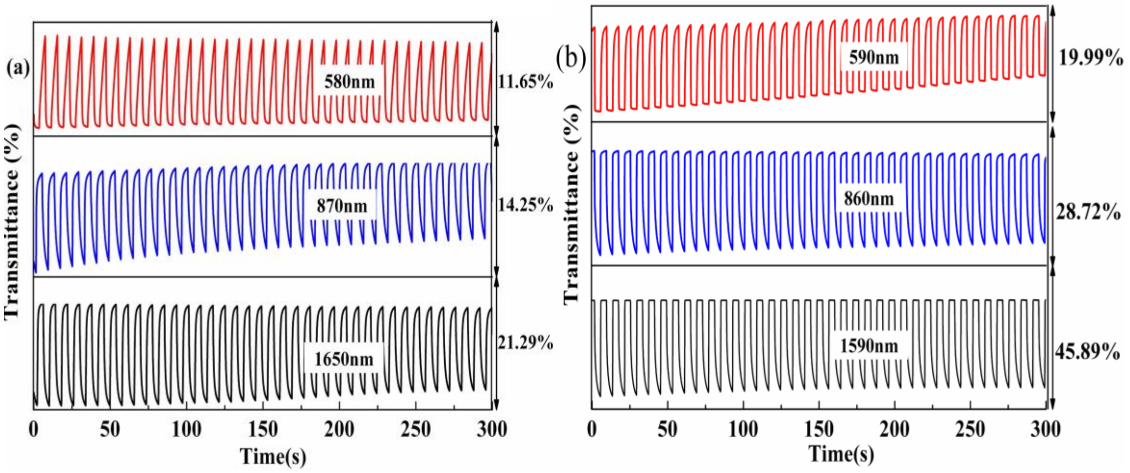

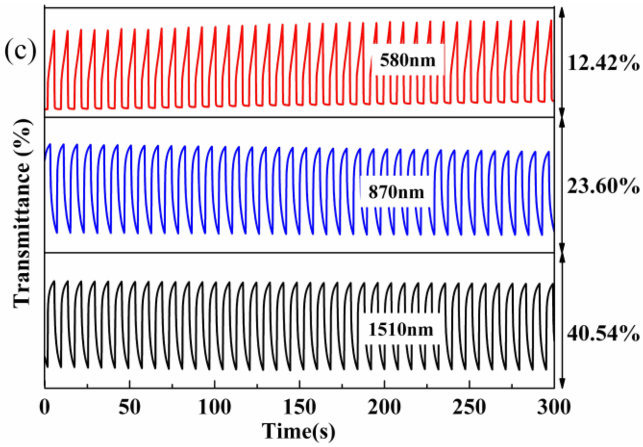

Figure S14. The chronoabsorptometry of three fluorene based polymers with the interval times of 5 s in the square wave potential method. The test wavelengths and the corresponding contrast ratios are labeled in the figures. (a), P(HT-BSe-OF); (b) P(HoT-BSe-OF); (c) P(OoT-BSe-OF).

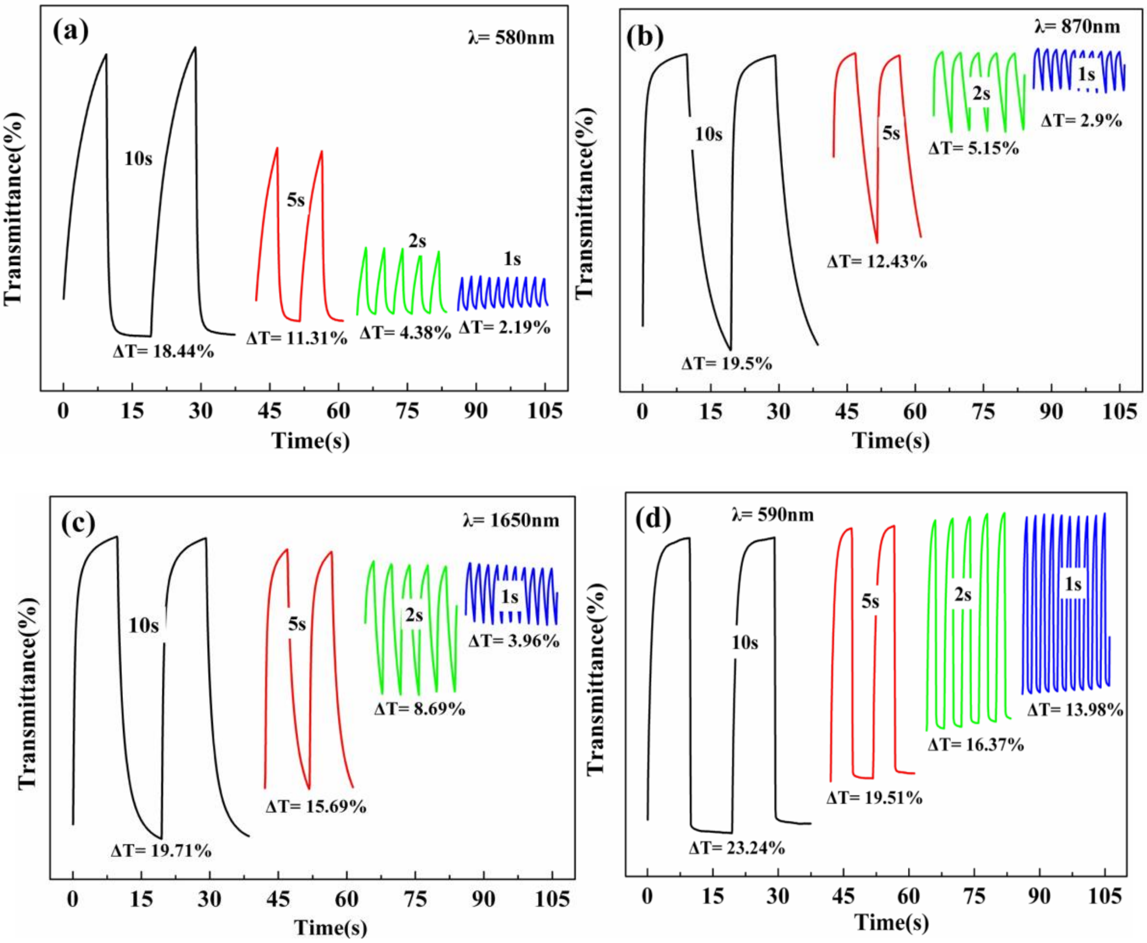

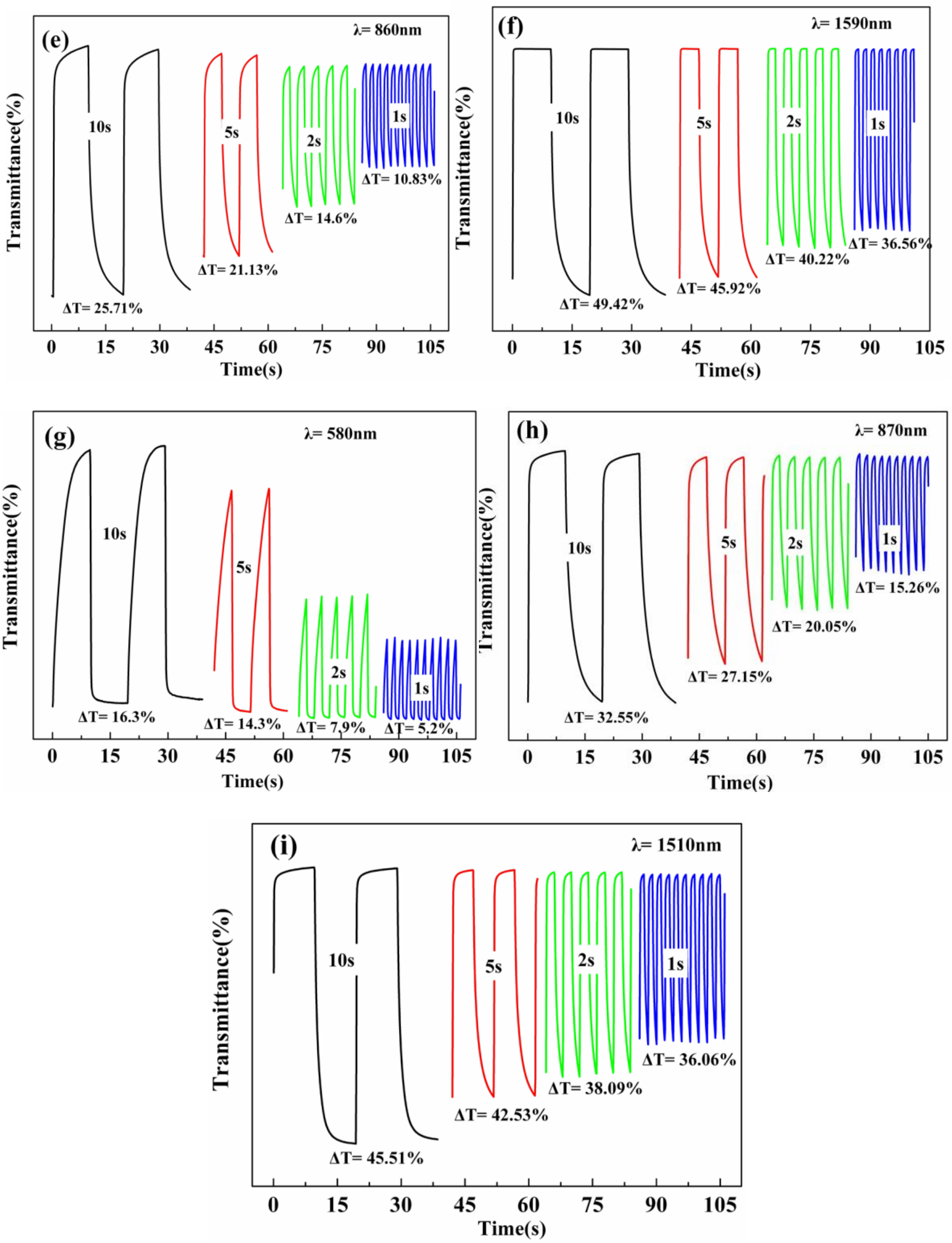

Figure S15. The dependence of the optical contrast ratios on the interval times in the chronoabsorptometry study. The interval times stetted in the in the square wave potential method varied at 10 s, 5 s, 2 s, 1 s in turn. The test wavelengths and the corresponding contrast ratios are labeled in the figures. (a-c), P(HT-BSe-OF); (d-f): P(HoT-BSe-OF); (g-i): P(OoT-BSe-OF).

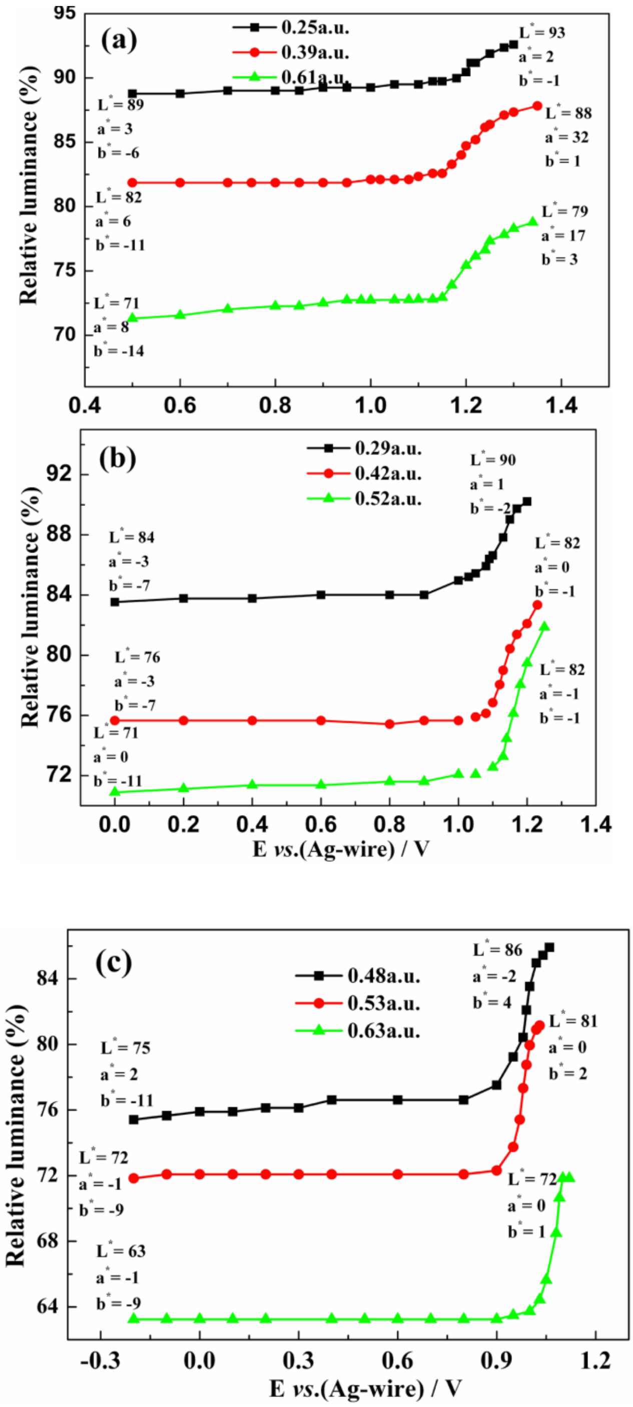

Figure S16. Relative luminance of polymer films as function of the externally applied potentials for three carbazole based copolymers. (a) P(HT-BSe-OF); (b) P(HoT-BSe-OF); (c) P(OoT-BSe-OF).

© 2018 by the authors. Submitted for possible open access publication under the terms and conditions of the Creative Commons Attribution (CC BY) license (http://creativecommons.org/licenses/by/4.0/).
